# AKT/protein kinase B associates with β-actin in the nucleus of melanoma cells

**DOI:** 10.1042/BSR20181312

**Published:** 2019-01-30

**Authors:** Larissa Leggieri Coa, Thiago Ferreira Abreu, Alexandre Keiji Tashima, Janaina Green, Renata Castiglioni Pascon, Marcelo Afonso Vallim, Joel Machado-Jr

**Affiliations:** 1 Department of Biological Sciences, Universidade Federal de São Paulo – UNIFESP, Diadema - SP, Brazil; 2 Department of Biochemistry, Escola Paulista de Medicina, Universidade Federal de São Paulo - UNIFESP, São Paulo - SP, Brazil

**Keywords:** actin, cofilin, mass spectrometry, protein-protein interactions, protein kinase B

## Abstract

The serine-threonine kinase AKT/PKB is a critical regulator of various essential cellular processes, and dysregulation of AKT has been implicated in many diseases, including cancer. Despite AKT action is known to function mainly in the cytoplasm, AKT has been reported to translocate to the nucleus. However, very little is known about the mechanism required for the nuclear import of AKT as well as its function in this cellular compartment. In the present study, we characterized the presence of endogenous nuclear AKT in human melanoma cells and addressed the possible role of AKT by exploring its potential association with key interaction nuclear partners. Confocal and Western blot analyses showed that both phosphorylated and non-phosphorylated forms of AKT are present in melanoma cells nuclei. Using mass spectrometry in combination with protein-crosslinking and co-immunoprecipitation, we identified a series of putative protein partners of nuclear AKT, including heterogeneous nuclear ribonucleoprotein (hnRNP), cytoskeleton proteins β-actin, γ-actin, β-actin-like 2 and vimentin. Confocal microscopy and biochemical analyses validated β-actin as a new nuclear AKT-interacting partner. Cofilin and active RNA Polymerase II, two proteins that have been described to interact and work in concert with nuclear actin in transcription regulation, were also found associated with nuclear AKT. Overall, the present study uncovered a yet unrecognized nuclear coupling of AKT and provides insights into the involvement of AKT in the interaction network of nuclear actin.

## Introduction

The serine-threonine kinase AKT (also named protein kinase B, PKB) is a critical regulator of various essential physiological cellular processes including cell proliferation, survival, motility, metabolism and differentiation [1]. Dysregulation of AKT pathway has been implicated in many diseases, including cancer [2]. It is well established that a multi-step process activates AKT, comprising a cascade of events that depend on PtdIns-3-4-5-P_3_ (PIP_3_), which are products of phosphoinositide 3-kinase (PI3K). Interaction of PIP_3_ with the pleckstrin homology (PH) domain of AKT brings AKT close to the plasma membrane and leads to a conformational change, which converts AKT as a substrate for regulatory kinases such as phosphoinositide-dependent kinase 1 (PDK-1) that phosphorylates AKT at Thr^308^. Phosphorylation at Ser^473^ is also required for maximal AKT activation, which is regulated by the mammalian target of rapamycin complex (mTORC2) [3]. The tumor suppressor lipid phosphatase PTEN, which is frequently mutated or deleted in various human cancers, antagonizes PI3K signaling by dephosphorylating PIP_3_ to PIP_2_ [4]. Once phosphorylated and active, AKT translocates from the inner surface of the cell membrane to other cell compartments to interact and phosphorylate a wide spectrum of substrates that regulate a diversity of cellular process [5].

Despite AKT action is acknowledged to function mainly in the cytoplasm, AKT has been reported to translocate to the nucleus of various cell types [6]. In this context, AKT has been found to enter the nucleus in response to different stimuli such as serum, insulin-like growth factor-1 (IGF-1), insulin, nerve growth factor (NGF) as well as F(ab’)2 fragment of anti-mouse IgG on B-cell receptor [7–11]. Accumulation of nuclear AKT has also been described in cancers such as lung, breast, thyroid, prostate and melanoma [12–16]. In fact, nuclear AKT was reported to increase during the progression of prostatic neoplasia [14] and has been associated with invasion and metastasis in human thyroid cancer [15]. Also, it has been shown that the promyelocytic leukemia tumor suppressor (PML) impair cancer progression by dephosphorylating and inactivating nuclear AKT [17], indicating that AKT residing in the nucleus plays a role in tumor development. However, many questions regarding the molecular mechanisms underlying the cytoplasmic-nuclear transport of AKT still unanswered. For example, AKT lacks a nuclear localization signal (NLS) motif, suggesting that the mechanism that governs the access of AKT to the nucleus is achieved through association with a yet unknown protein [6]. Moreover, still unclear whether phosphorylation of AKT is necessary for its nuclear translocation. Both non-phosphorylated and phosphorylated AKT have been described to exist at the nuclear compartment and these discrepancies seem to depend on cell type and/or stimuli used [6]. Yet, it has been demonstrated that the nucleus encloses several components of the AKT signaling, such as PI3K, PIP3, PDK1 and mTORC2 [18–20], suggesting that nuclear AKT might be phosphorylated directly within this compartment.

The vast array of AKT functions on cellular responses is achieved through direct interaction with specific substrates as well as with non-substrate proteins. Some progress has been made in the identification of AKT substrates in the nucleus, among them regulators of the cell cycle such as p21 and p27 [21], proteins involved in cell survival like Acinus [22], Prohibitin 2 [23] and the transcription factor Forkhead/FOXO [24]. Also, non-substrate proteins such as Ebp1 [25] and B23/NPM [26] have been identified to interact with nuclear AKT resulting in the regulation of cell survival. Thus, identification of additional interacting proteins of nuclear AKT may provide a better understanding of how this kinase modulates signaling in the nucleus that is distinct from its correspondent cytosolic subset.

In the present study, we identified and characterized the presence of endogenous AKT in the nucleus of the human melanoma A2058 cell line. Using co-immunoprecipitation (Co-IP) combined with cross-linking and mass spectrometry, we found a series of putative protein partners of nuclear AKT. Among them, we validated β-actin as a new nuclear AKT-interacting partner. We also showed evidence that Cofilin and active RNA Polymerase II, two proteins that have been described to interact and work in concert with nuclear actin in the regulation of gene transcription, were also found associated with nuclear AKT. Overall, our findings provide insights into the involvement of AKT in the interaction network of nuclear actin.

## Materials and methods

### Cell lines and reagents

The human melanoma cell line A2058 (mutation status: BRAF V^600E^ [27], PTEN mutated [28]) were cultured in RPMI 1640 medium supplemented with 10% fetal bovine serum (FBS), 1% penicillin and streptomycin, and 1% l-glutamine (Invitrogen). Cell culture experiments were performed at 37°C and 5% CO_2_. During growth factor deprivation experiments, FBS was omitted from the medium.

### Antibodies and inhibitors

Primary antibodies: anti-AKT-PAN (C67E7), anti-p-AKT-473 (D9E), anti-p-AKT-308 (C31E5E), anti-CREB (86B10), anti-Cofilin (D3F9), all from Cell Signaling Technology, Inc. Anti-β-Actin (A2228) was from Sigma-Aldrich and anti-RNA polymerase II CTD repeat YSPTSPS (phospho S2) antibody (ab24758) was from Abcam. Fluorescent dye-conjugated and HRP-conjugated secondary antibodies were all from Cell Signaling Technology, Inc. PI3K inhibitor-XI, a cell-permeable Wortmannin 17β-Hydroxy analogue (HWT) that acts as an irreversible PI3K inhibitor (EMD Millipore #58053-83-1).

### Cytoplasmic/nuclear protein extraction and immunoblotting

Nuclear and cytoplasmic extracts were isolated as described previously [29]. Briefly, cells were lysed in a hypotonic buffer (10 mM Hepes, pH 7.9, 60 mM KCl, 1 mM EDTA, 1 mM DTT, 0.5% NP-40) containing protease and phosphatase inhibitors (Sigma-Aldrich). The nuclei were pelleted by centrifugation at 500 ***g*** for 5 min at 4°C and the supernatants (cytoplasmic extract) were collected. Nuclei were washed twice in the hypotonic buffer without NP-40 and then ressuspended in a Tris-HCl buffer (250 mM Tris-HCl, pH 7.8, 60 mM KCl, 1 mM EDTA, 1 mM DTT, 0.5% NP-40) containing protease and phosphatase inhibitors at the same concentration as in the hypotonic buffer. Nuclear membranes were disrupted by freeze-thawing followed by centrifugation at 15000 ***g*** for 30 min to remove any trace of membrane structures. The supernatants (nuclear extracts) were collected and either used immediately or stored at –80°C until use. For immunoblotting, nuclear and cytoplasmic extracts were separated by SDS-PAGE, transferred to PVDF membranes and immunoblotted using 50 μg of cell lysate. Blots were processed for enhanced chemiluminescence (Pierce) and immunoreactive bands visualized and quantified using Uvitec Alliance 4.7 Cambridge®.

### Two-step chemical cross-linking and immunoprecipitation

Cross-linking and co-IP procedures were executed as described elsewhere with minor modifications [30]. Briefly, for binding of the specific antibody to Protein A/G agarose, Protein A/G agarose slurry (Sigma-Aldrich) was washed twice with 200 μl PBS buffer and incubated with 100 μl antibody prepared in PBS (10 μl antibody + 8.5 μl H_2_O + 5 μl 20× PBS) at 25°C for 30 min on a mixer. As a negative control, the same procedure was done using anti-rabbit or anti-mouse IgG peroxidase secondary antibody (depending on the specificity of the experimental antibody used). The supernatant was discarded and the beads were washed three times with 300 μl PBS, followed by incubation with succinimidyl suberate (DSS) solution (2.5 μl 20× PBS + 38.5 μl H_2_O + 2.5 mM DSS in DMSO) at 25°C for 45 to 60 min on a mixer. After removing the supernatant, the beads were washed three times with 50 μl 100 mM glycine (pH 2.8), twice with PBS containing 1% NP-40, then once with 300 μl PBS. The antibody-crosslinked beads were incubated with 500 μg nuclear lysates of melanoma cells overnight at 4°C on a shaker. The incubation continued after adding 20 μl 50 nM dithiobis[succinimidylpropionate] (DSP) in DMSO for 2 h. The DSP-crosslinking was quenched with 30 μl 1 M Tris-HCl pH 7.4 (30 min). After removing supernatant and washing five times with 300 μl washing buffer (25 mM Tris, 150 mM NaCl, 1 mM EDTA, 1% NP-40, 5% glycerol, pH 7.4), the co-immunoprecipitation product was clued with 40 μl 2× Laemmli buffer at 100°C for 10 min. The eluting complex was subjected to SDS-PAGE separation for immunoblotting or MS/MS analysis.

### In-gel digestion

AKT co-immunoprecipitated material from nuclear extracts of melanoma cells was loaded onto a 10% Bis-Tris gel and submitted to electrophoresis at a constant voltage of 50 V. The separated proteins were visualized by Coomassie blue staining. Bands were excised and processed for in-gel trypsin digestion. Gel pieces were destained with 50 mM NH_4_HCO_3_ in 50% acetonitrile (Sigma-Aldrich), dried by vacuum centrifugation and incubated with 100 μl of 10 mM DTT and 50 mM NH_4_HCO_3_ for 1 h at 56°C for disulfide bond reduction. Samples were subjected to in-gel cysteine alkylation with 100 μl of 55 mM iodoacetamide (Sigma-Aldrich) in 50 mM NH_4_HCO_3_ at room temperature for 45 min on dark. After two sequential washes with 200 μl of 50 mM NH_4_HCO_3_ in 50% acetonitrile gel, pieces were dried and rehydrated with 12.5 ng/μl trypsin gold (Promega) solution in 50 mM NH_4_HCO_3_ for 15 min on ice. The digestion was continued overnight at 37°C. The tryptic peptides were extracted with 5% formic acid/50% acetonitrile at room temperature for 45 min on a shaker. The supernatant was stored and the gel pellet was submitted to a second round of extraction with 5% formic acid/100% acetonitrile for 45 min at room temperature. The supernatants from both rounds of extraction were pulled together, concentrated with vacuum centrifugation and desalted using ZipTip C-18 (Millipore).

### Mass spectrometric analysis

The LC-MS^E^ experiments were performed on a Synapt G2 mass spectrometer coupled to a nanoAcquity UPLC system (Waters, Milford, MA, U.S.A.). The peptide mixture was loaded online for 5 min at a flow rate of 8 µl/min of phase A (0.1% formic acid) using a Symmetry C_18_ trapping column (5 µm particles, 180 µm x 20 mm length; Waters, Milford, MA, U.S.A.). The mixture of trapped peptides was subsequently separated by elution with a gradient of 7 to 35% of phase B (0.1% formic acid in acetonitrile) through a BEH 130 C18 column (1.7 µm particles, 75 × 150 mm; Waters, Milford, MA, U.S.A.) in 12 min, at 275 nl/min. Data were acquired in the data-independent mode in the *m/z* range of 50 to 1600 in resolution mode. Collision energies were alternated between 4 eV and a ramp of 17 to 45 eV for precursor ion and fragment ions, respectively, using scan times of 1.25 s. The ESI source was operated in positive mode with a capillary voltage of 3.0 kV, block temperature of 70°C and cone voltage of 50 V. For lock mass correction, [Glu^1^]-Fibrinopeptide B solution (500 fmol/ml in 50% acetonitrile, 0.1 formic acid; Waters, Milford, MA, U.S.A.) was infused through the reference sprayer at 500 nl/min and sampled every 30 s. Analyses were run in duplicates. MS data were processed by ProteinLynx Global Server v3.0.1 using low energy threshold of 150 counts, elevated energy threshold of 30 counts and intensity threshold of 750 counts. The MS/MS spectra were exported as a *.pkl file and imported in PEAKS Studio 7.5 (Bioinformatics Solution Inc., Waterloo, Canada) [31] for *de novo* analysis and database search. *De novo* analysis was carried out with precursor mass tolerance of 10 ppm, fragment mass tolerance of 0.05 Da and trypsin as the digesting enzyme allowing up to two missed cleavages. Cys carbamidomethylation was set as fixed modification and Met oxidation was set as variable modification. *De novo* sequenced peptides with average local confidence (ALC) scores ≥50% were selected for database searches against the human database from UniProtKB (20,269 sequences, downloaded in August 21, 2013 from www.uniprot.org). The peptide false discovery rate was estimated by the decoy fusion method [31] and was set at a maximum of 1%.

### Immunofluorescence and confocal microscopy

For immunofluorescence studies, cells were grown on glass coverslips, washed in PBS and fixed in 4% paraformaldehyde-PBS solution for 20 min at room temperature. Cells were then permeabilized for 10 min in PBS containing 0.2% Triton X-100, washed in PBS and then blocked for 30 min at room temperature with blocking buffer containing 10% goat serum in PBS. After blocking, slides were incubated overnight with 1:50 dilution of the corresponding primary antibody, washed extensively with blocking buffer and stained with the appropriate AlexaFluor-conjugated secondary antibody for 1 h at room temperature. Samples were washed extensively and mounted with Prolong antifade containing DAPI (Invitrogen). For double immunofluorescence staining, secondary antibodies were properly checked to avoid cross-reaction with isotype or species of the primary antibody immunoglobulin. Confocal images were acquired with a Leica TCS SP8 laser-scanning microscope (Leica, Germany). The series of images obtained from confocal z-stacks and orthogonal plane reconstruction were processed and analyzed using ImageJ software. Co-localization analysis was performed using the Colocalization Threshold plugin for ImageJ [32], which generates a merged image with white areas displaying the co-localization of two signals.

## Results

### Identification and characterization of endogenous AKT in the nucleus of melanoma cells

**Figure 1:**
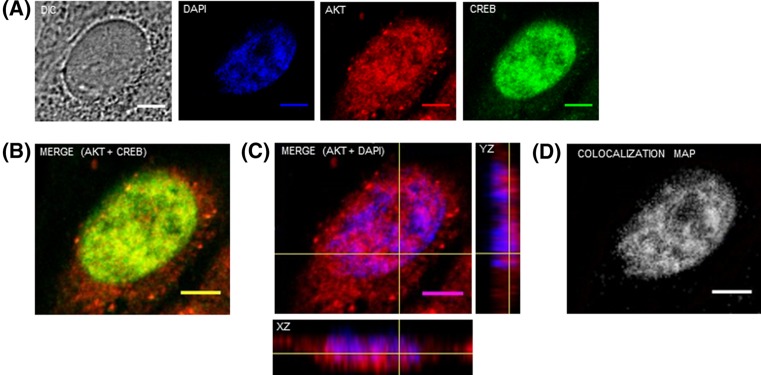
AKT is localized in the nucleus of melanoma cells A2058 cells were double immunostained for AKT and CREB and analyzed by confocal laser-scanning microscopy. Images are projections of one stack from the middle plane of the nucleus. (**A**) AKT is shown in red, CREB is shown in green, DAPI-stained nuclei are shown in blue and DIC represents differential interference contrast image. (**B**) Merged image of endogenous AKT and CREB. (**C**) Merged image of AKT and DAPI. The yellow cross in the merged panel of AKT and DAPI shows the confocal *z*-series reconstruction and corresponding *xz* and *yz* orthogonal planes. Confocal image and orthogonal views show that AKT immunostaining is localized in melanoma cells nuclei. (**D**) Co-localization map for AKT and DAPI, obtained using the Colocalization Threshold plugin for ImageJ, shows pixels with positive signals for both probes in white; scale bar, 5 μm.

First, we determined the nuclear localization of endogenous AKT by confocal immunofluorescence. Therefore, A2058 cells were stained with antibodies that specifically recognize AKT. We also marked the transcription factor CREB as a positive control for nuclear localization, since this protein is constitutively present in the nucleus of different cell types. Immunofluorescence analysis by confocal laser scanning microscopy (Figure 1A) showed that CREB (green) is localized predominantly in the nucleus while AKT (red) is present in the cytoplasm but also within the nucleus of A2058 cells. The merged images indicate co-localization of AKT+CREB (Figure 1B) and AKT+DAPI (violet) (Figure 1C). Lines on the merged projection image of AKT+DAPI indicate the vertical (*YZ*) and horizontal (*XZ*) cross-sections from Z-stack reconstruction, showing that AKT is found within the nucleus. Co-localization of AKT with DNA (DAPI) was also confirmed using the Colocalization Threshold plugin for ImageJ, which generates a merged image with white pixels displaying the co-localization of both probes (Figure 1D).

**Figure 2:**
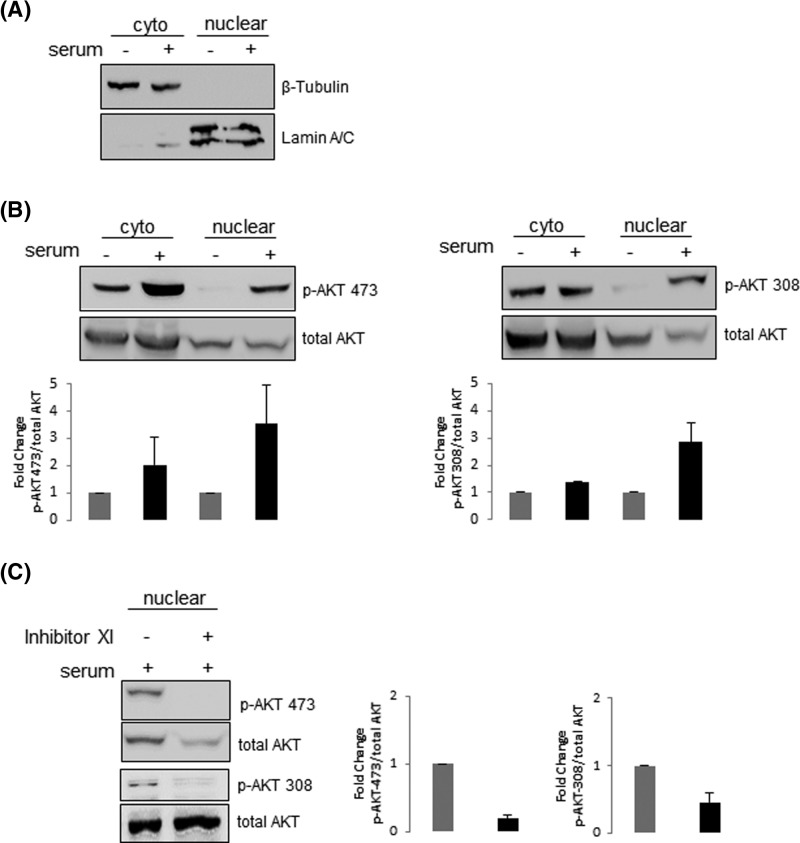
Both phosphorylated and non-phosphorylated AKT are present in melanoma cells nuclei A2058 cells were serum starved for 24 h and then stimulated (+) or not (−) with 10% serum for 30 min. (**A**) Nuclear and cytoplasmic fractions were analyzed by Western blot with anti-lamin A/C or anti-β-tubulin to confirm the purity of the nuclear and cytoplasmic fractions, respectively. (**B**) Nuclear and cytoplasmic fractions were analyzed by Western blot with anti-p-AKT-Ser^473^, anti-p-AKT-Thr^308^ and anti-total-AKT. (**C**) A2058 cells were serum starved for 24 h in the presence (+) or absence (−) of the PI3K inhibitor XI (20 μM) and then stimulated with 10% serum for 30 min (+). Nuclear fractions were analyzed by Western blot with anti-p-AKT-Ser^473^, anti-p-AKT-Thr^308^ and anti-total-AKT. (B and C) Results were plotted as the mean +/− S.D., *n*=3. Fold change in AKT phosphorylation was normalized to the levels of total-AKT.

To further characterize nuclear AKT in A2058 cells, we examined the phosphorylation status of AKT localized both in the cytoplasm and nucleus. Therefore, A2058 cells were serum starved for 24 h and then stimulated or not with serum for 30 min. Nuclear and cytoplasmic extracts were produced and submitted to immunoblotting for the detection of AKT phosphorylated either on Ser^473^ or Thr^308^. Western blotting using anti-lamin A/C or anti-β-tubulin confirmed the purity of the nuclear and cytoplasmic fractions, respectively (Figure 2A). As expected, constitutive AKT phosphorylation on Ser^473^ and Thr^308^ was high levels in the cytoplasm, consistent with PTEN deficiency in A2058 cells (Figure 2B). Following serum treatment, phosphorylation of cytoplasmic AKT-Ser^473^ increased one fold compared with unstimulated cells, while a less prominent increase was observed for p-AKT-Thr^308^. In nuclear fractions, p-AKT Ser^473^ and Thr^308^ showed reduced levels in unstimulated cells, which were enhanced upon stimulus, suggesting that the accumulation of p-AKT in the nucleus is favored in the presence of serum. However, when membranes were stripped and reprobed with anti-total-AKT, which recognizes AKT irrespective of its phosphorylation state, total-AKT showed similar expression levels in the nucleus irrespective of serum stimulation (Figure 2B). Since p-AKT was barely undetected in unstimulated cells in contrast with total-AKT, these observations suggest that AKT is also present in the nucleus of unstimulated cells but most likely in its non-phosphorylated form (Figure 2B). To check this point, we asked whether inhibition of serum-increased AKT phosphorylation would disturb AKT nuclear localization. If phosphorylation is critical for AKT nuclear residence, inhibition of nuclear AKT phosphorylation should display reduced levels of total-AKT as well. Thus, A2058 cells were serum-starved in the presence or absence of the PI3K inhibitor XI and then stimulated with serum. As shown in Figure 2C, while serum-increased nuclear AKT phosphorylation on both residues was repressed by the PI3K inhibitor XI, total-AKT still expressed, suggesting that both phosphorylated and non-phosphorylated AKT are present in melanoma cells nuclei.

### Identification of binding partners of Nuclear AKT

**Figure 3:**
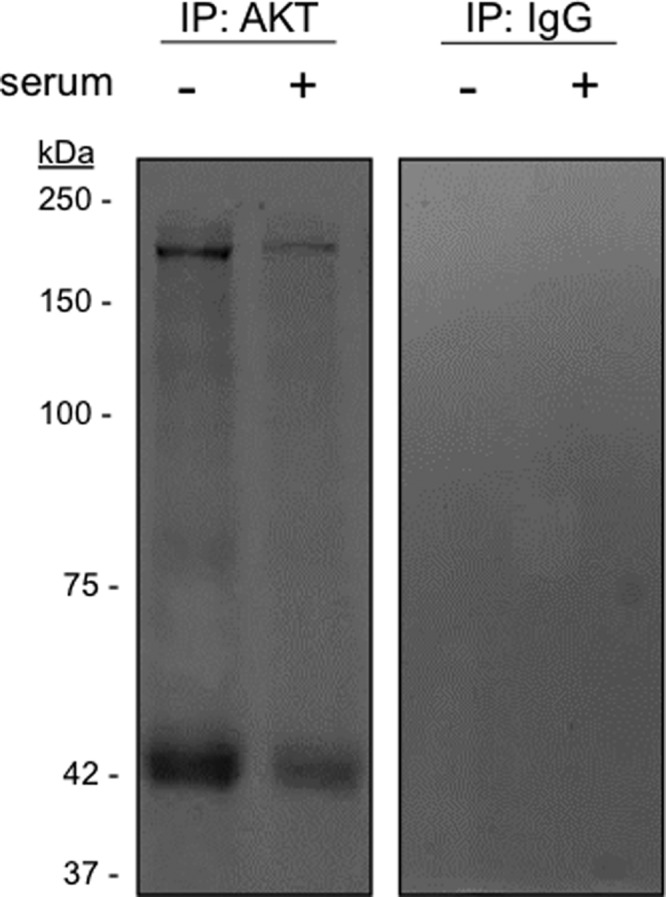
Identification of nuclear AKT binding proteins in melanoma cells nuclei A2058 cells were serum starved for 24 h and then stimulated (+) or not (−) with 10% serum for 30 min as indicated. Nuclear extracts were submitted to two-step chemical cross-linking and immunoprecipitations (IP) were performed using anti-total AKT or control IgG antibodies. Co-immunoprecipitated materials were resolved on SDS-PAGE. Coomassie blue staining of the gel revealed co-immunoprecipitated bands of approximately 42 and 200 kDa. The identified bands were excised and subjected to trypsin digestion for LC-MS/MS analysis.

In order to gain insight into the function of nuclear AKT, we performed a two-step chemical cross-linking and co-IP-based mass spectrometry to identify potential novel nuclear AKT interaction partners. This methodology is based on the immobilization of AKT antibody to protein A/G beads with the use of an irreversible cross-linker (DSS). A second cleavable cross-linker (DSP) was subsequently employed to produce stable complexes between AKT and associated proteins before SDS-PAGE and LC-MS/MS analysis. The predominant interacting nuclear proteins were identified by co-IP with anti-total AKT antibody by one-dimensional SDS-PAGE. Two co-immunoprecipitated bands with the size of 42 kDa and approximately 200 kDa were detected in nuclear extracts of A2058 cells irrespective of serum-stimulation, but not from the negative control where IgG was used instead of anti-AKT (Figure 3). To ensure reproducible identification of nuclear AKT partners from the identified bands at least two independent mass spectrometry analyses were performed. In addition, to restrict our search for nuclear AKT-binding partners, only proteins that were identified in at least two independent analyses were considered. Based on these criteria, we circumscribed our investigation considering exclusively proteins identified from the major band of 42 kDa. Our database examination documented several proteins as putative nuclear AKT-binding partners including heterogeneous nuclear ribonucleoprotein (hnRNP), a protein with known nuclear function, as well as cytoskeleton proteins β-actin, γ-actin, vimentin and β-actin like 2′ (Table 1).

**Table 1:** Nuclear AKT-interacting proteins identified by LC-MS/MS analysis of gel fragments derived after immunoprecipitation with anti-AKT

Cell line	Treatment	Access no.	PEAKS score	Coverage (%)	Unique peptides	MW	Protein name
A2058	unstimulated	P60709	189.9	29	12	42	β-Actin
		P22626	92.8	10	3	37	Heterogeneous nuclear ribonucleoprotein (hnRNP) A2/B1
	stimulated	P63621	167.6	28	7	42	γ-Actin
		Q562R1	173.3	18	10	42	β-actin-like 2
		P08670	118.8	17	8	54	Vimentin

Although cytoskeleton proteins have well-defined functions in the cytoplasm, there are numerous lines of evidence that different types of cytoskeleton proteins are localized to the nucleus, suggestive of their involvement in the regulation of nuclear functions [33]. In particular, there have been convincing data demonstrating that actin is not only present in the nucleus but also plays important roles in diverse nuclear complexes linked to gene expression such as transcription and chromatin remodeling [34]. In view of these premises, we considered actin as an attractive candidate and therefore its interaction with nuclear AKT was further investigated. A complete listing of all identified peptides sequences information and spectra from LC-MS/MS analysis are provided in Supplementary Table S1 and Figure S1, respectively (see additional files 1 and 2).

### Validation of nuclear AKT and actin interaction

**Figure 4:**
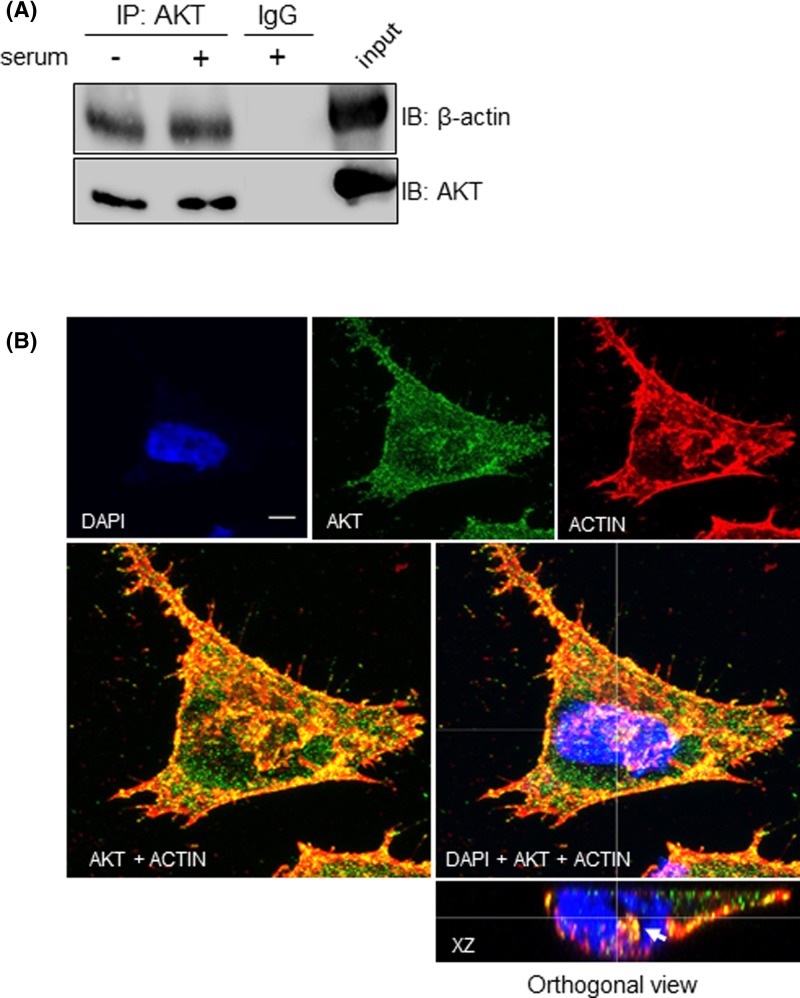
Nuclear AKT interacts with β-actin A2058 cells were serum starved for 24 h and then stimulated (+) or not (−) with 10% serum for 30 min, as indicated. (**A**) Nuclear lysates were submitted to immunoprecipitation (IP) with anti-total AKT or control IgG antibodies. Cell lysates prior to immunoprecipitation were used as input. Input and co-immunoprecipitated proteins were immunoblotted (IB) for detection of total AKT and β-actin. (**B**) A2058 cells were double immunostained for AKT and β-actin and analyzed by confocal laser-scanning microscopy. Confocal images show maximum projection of image stacks. AKT is shown in green, β-actin is shown in red, DAPI-stained nuclei are shown in blue. Merged images are shown at higher magnification in the panels below. Reconstructed orthogonal projection is presented as viewed in the *xz* plane, showing co-localization of AKT with β-actin in the nucleus (yellow pixels, arrowhead); scale bar, 5 μm.

To confirm the interaction between nuclear AKT and actin, we checked whether the β-actin isoform could be co-immunoprecipited with AKT. Therefore, AKT was immunoprecipitated from nuclear lysates, separated by SDS-PAGE, and immunoblotted with anti-β-actin and anti-AKT antibodies. As shown in Figure 4A, β-actin co-immunoprecipitated with nuclear AKT irrespective of serum stimulation, but not from the negative control where IgG was used instead of anti-AKT. In line with these results, analysis of confocal z-series reconstruction in corresponding *xz* orthogonal plane confirmed co-localization of AKT with β-actin in the nucleus (Figure 4B).

### Phosphorylation of AKT is not required for interaction with β-actin

**Figure 5:**
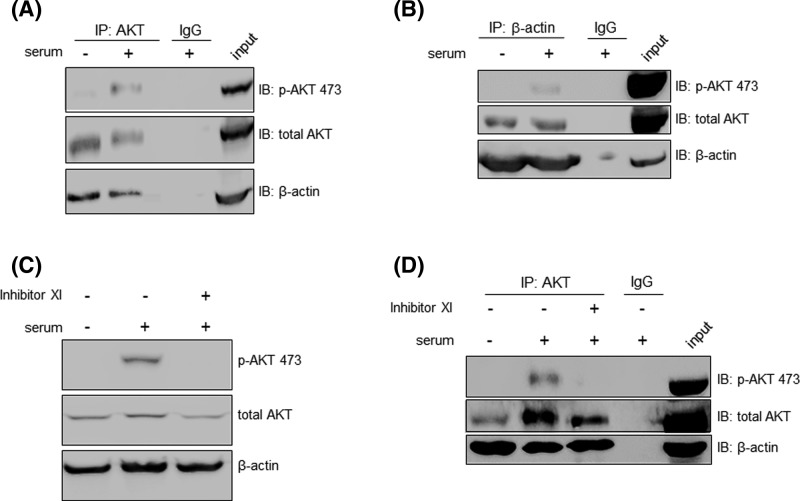
AKT/β-actin complex is not disturbed by inhibition of AKT phosphorylation (**A**) A2058 cells were serum starved for 24 h and then stimulated (+) or not (−) with 10% serum for 30 min as indicated. Nuclear fractions were immunoprecipitated (IP) with anti-total AKT or control IgG antibodies. Cell lysates prior to immunoprecipitation were used as input. Input and co-IP proteins were resolved on SDS-PAGE and immunoblotted for the detection of p-AKT Ser^473^, total AKT and β-actin (**B**). The result shown in (A) was validated by reverse IP where anti-β-actin was used for immunoprecipitation instead of anti-total AKT. (**C**) A2058 cells were serum starved for 24 h in the presence (+) or absence (−) of the PI3K inhibitor XI (20 μM) and then stimulated (+) or not (−) with 10% serum for 30 min. Nuclear fractions were analyzed by Western blot with anti-p-AKT Ser^473^, anti-total AKT and anti-β-actin. (**D**) A2058 cells were treated as described in C and nuclear lysates were immunoprecipitated with anti-total AKT or control IgG antibodies. Co-IP proteins were resolved on SDS-PAGE and immunoblotted for detection of p-AKT Ser^473^, total AKT and β-actin.

To investigate the interaction of nuclear AKT and β-actin in a more functional setting, we checked whether AKT phosphorylation status could affect AKT/β-actin complex formation. Therefore, A2058 cells were stimulated or not with serum and nuclear lysates were immunoprecipitated with anti-AKT. As shown in Figure 5A, β-actin co-immunoprecipitated with AKT despite serum-induced AKT Ser^473^ phosphorylation, suggesting that both phosphorylated and non-phosphorylated nuclear AKT interact with β-actin. These results were also corroborated by reverse immunoprecipitation where anti-β-actin was used as bait, instead of anti-AKT (Figure 5B). To confirm that phosphorylation of AKT is not required for interaction with β-actin, we examined whether recruitment of β-actin by nuclear AKT was sensitive to PI3K inhibition. As shown in Figure 5C, serum-induced nuclear AKT-Ser^473^ phosphorylation was repressed by the PI3K inhibitor XI without affecting β-actin levels in the nucleus. By co-IP analysis we demonstrated that AKT/β-actin coupling was not disturbed by inhibition of AKT phosphorylation, since β-actin was still interacting with AKT in the presence of the PI3K inhibitor XI, suggesting that nuclear AKT/β-actin complex is formed regardless of AKT-Ser^473^ phosphorylation state (Figure 5D).

### Nuclear AKT interacts with components of the transcriptional machinery

**Figure 6:**
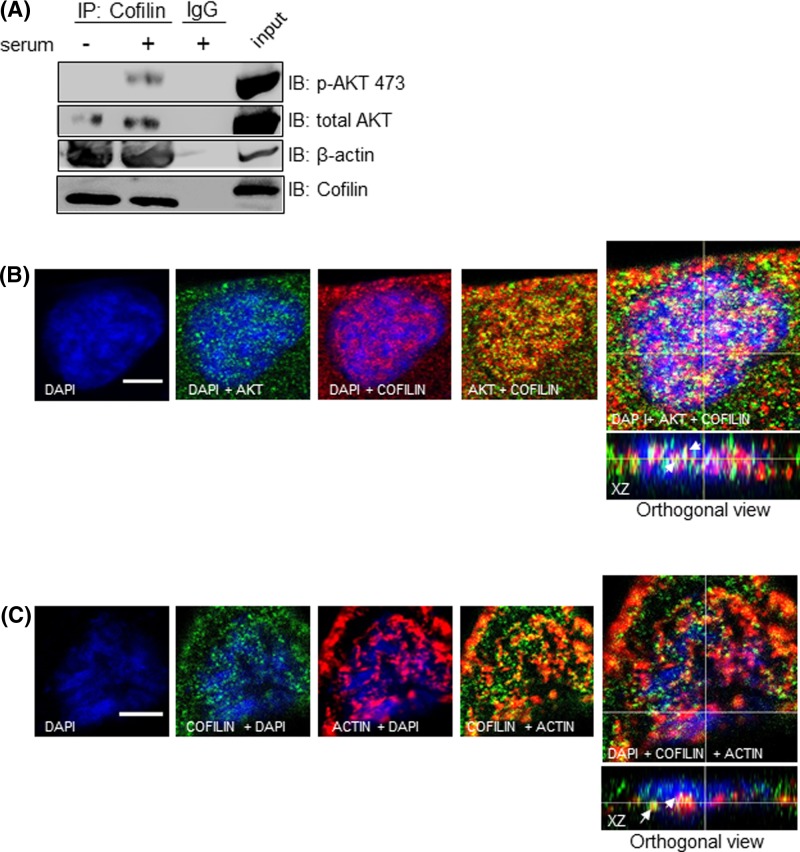
Nuclear AKT interacts with cofilin (**A**) A2058 cells were serum starved for 24 h and then stimulated (+) or not (−) with 10% serum for 30 min, as indicated. Nuclear fractions were immunoprecipitated (IP) with anti-cofilin or control IgG antibodies. Cell lysates prior to immunoprecipitation were used as input. Input and co-IP proteins were resolved on SDS-PAGE and immunoblotted for the detection of p-AKT^473^, total AKT, β-actin and cofilin. (**B** and **C**) A2058 cells were double immunostained for: (B) AKT (green) and Cofilin (red); (C) cofilin (green) and β-actin (red). DAPI-stained nuclei are shown in blue. Immunostained cells were analyzed by confocal laser-scanning microscopy. Images are projections of one stack from the middle plane of the nucleus. Reconstructed orthogonal projections are presented as viewed in the *xz* planes, showing co-localized immunostaining in the nucleus (yellow pixels, arrowhead); scale bar, 5 μm.

**Figure 7:**
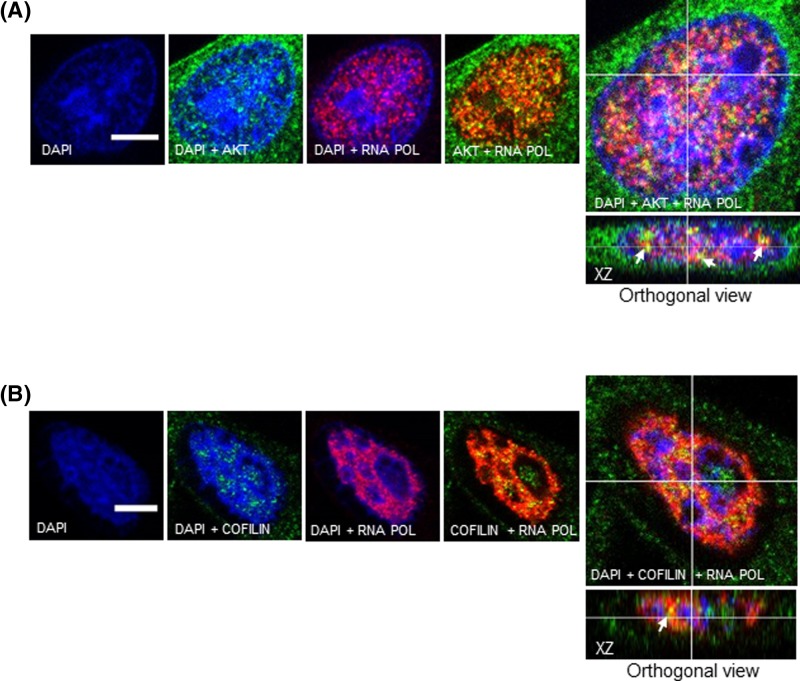
Both nuclear AKT and cofilin co-localize with active RNA Pol II A2058 cells were double immunostained either for (**A**) AKT or (**B**) Cofilin with RNA Pol II phosphorylated at Ser2 in the CTD. AKT and Cofilin are shown in green, RNA Pol II is shown in red and DAPI-stained nuclei are shown in blue. Immunostained cells were analyzed by confocal laser-scanning microscopy. Images are projections of one stack from the middle plane of the nucleus. Reconstructed orthogonal projections are presented as viewed in the *xz* plane, showing co-localized immunostaining in the nucleus (yellow pixels, arrowhead); scale bar, 5 μm.

It has been described that nuclear actin associated with the serine2/5- and Ser2-phosphorylated RNA polymerase II (Pol II) carboxy-terminal domain (CTD) is required for transcription elongation by Pol II [35]. Moreover, increasing evidence suggest that actin-binding proteins (ABPs) such as cofilin also associates with phosphorylated Pol II via interaction with nuclear actin, facilitating association of elongating Pol II and actin with active genes [36]. This information led us to ask whether nuclear AKT could be assembled with cofilin as well as with phosphorylated Pol II, providing insight into the spatial relationship of nuclear AKT with components that operate with actin in the transcription machinery. To examine this, we first performed co-IP experiments from nuclear extracts using anti-cofilin as bait. As shown in Figure 6A, both AKT and β-actin co-immunoprecipitated with cofilin. In addition, analysis of confocal z-series reconstruction in corresponding *xz* orthogonal planes showed that cofilin co-localizes with both AKT and β-actin at the nuclear compartment (Figure 6B,C). In order to assess if AKT and cofilin are located at sites of transcription, we performed confocal microscopy analysis to examine whether these proteins co-localize with Pol II phosphorylated at Ser2 in the CTD. As shown in Figure 7A,B, confocal z-series reconstruction in corresponding *xz* orthogonal planes showed that both nuclear AKT and cofilin co-localize with phospho-Pol II, suggesting that nuclear AKT and cofilin co-habit with Pol II to sites of active transcription.

## Discussion

Spatial dynamics of signaling proteins has been suggested to be of outstanding importance for cellular biological functions [37]. According to their subcellular localization, the same repertoire of signaling proteins can gain access to selected biochemical circuits of interacting partners, resulting in functional biological diversity. In this work, we explored this general principle of intracellular localization to uncover the interaction networks of AKT in the nucleus of melanoma cells.

We demonstrated that AKT is endogenously distributed both in the nucleus and cytoplasm of A2058 melanoma cells. We also showed evidence that phosphorylated and non-phosphorylated forms of AKT are present in melanoma cells nuclei, suggesting that phosphorylation is not critical for the nuclear retention of AKT. Although further work is needed to particularly understand the mechanism underlying AKT nuclear trafficking in melanoma cells, the literature highlight controversial reports on whether AKT nuclear translocation is dependent on its kinase activity or phosphorylation state. For instance, it has been demonstrated that non-phosphorylated AKT was found in the nucleus in HEK293 cells overexpressing inactive forms of AKT (K179M and T308A/S473A) that could not be phosphorylated [38]. On the other hand, the expression of the same mutants described above were not detected in the nucleus in PC12 cells stimulated with NGF [39], suggesting that this contrasting findings depend on cell-type and stimulus-specific signaling mechanisms. Also, convincing date from Wang and Brattain [40] showed that endogenous AKT was able to be phosphorylated and activated inside the nucleus of MCF7E cells in response to IGF-1. In fact, it has been demonstrated that the nucleus encloses several components of the AKT activation machinery such as PI3K, PI3P, PDK1 and mTORC2 [18–20,41], indicating that the interaction of AKT with these signaling components may be as dynamic in the nucleus as it is already known in the cytoplasm. Moreover, besides the importance whether AKT needs to be phosphorylated or not for entering the nucleus, it is conceivable that AKT is unable to do this by itself. The nuclear localization sequence (NLS) motif of AKT is unknown, indicating that a carrier protein is involved in its nuclear transport. Although some studies have pointed some attractive candidates for mediating the nuclear transport of AKT [42,43], this mechanism still inconclusive.

In the second phase of the present study, we performed a two-step chemical cross-linking strategy for co-IP followed by MS/MS-based analysis as a tool for mapping potential nuclear AKT binding partners. We identified a number of proteins as putative nuclear AKT partners and using a combination of biochemical and confocal imaging, we confirmed β-actin as a new nuclear AKT associate. The interaction between AKT and actin is not without precedents. Two different groups have demonstrated it. Cenni et al. [44] using recombinant proteins *in vitro* binding and overlay assays established that AKT interacts with actin directly through the N-terminal PH domain of AKT. Vandermoere et al. [45] analyzed proteins co-immunoprecipitated with AKT in MCF-7 breast cancer cells and by mass spectrometry and biochemical approaches showed that AKT not only interacts with actin but also is able to phosphorylate it, suggesting that actin is a substrate of AKT kinase activity. It should be noted, however, that subcellular fractionation were not employed in the works mentioned above and therefore, specific subcellular sites of interaction were not addressed. To the best of our knowledge, our study provides the first evidence that AKT and actin interact inside the nucleus.

**Figure 8:**
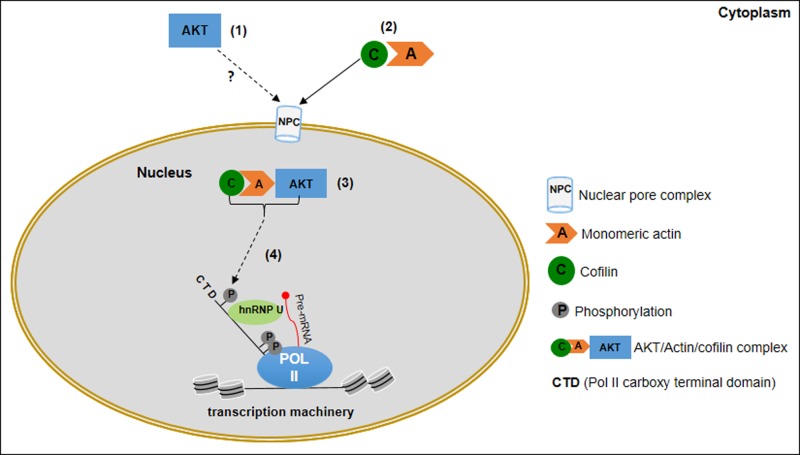
Schematic representation summarizing the interaction network of nuclear AKT and its predicted location at functional nuclear regions (**1**) AKT is translocated from the cytoplasm to the nucleus by an yet unknown mechanism; (**2**) cofilin bound to monomeric actin mediates the transport of actin to the nucleus; (**3**) In the nucleus, AKT interacts with actin/cofilin complex; (4) AKT interaction with actin/cofilin complex may be target to active Pol II phosphorylated in the CTD.

Nuclear actin has gained great attention in recent years for its role in several processes that regulate gene expression [34]. It is associated with and required for transcription by all eukaryotic RNA polymerases [46–49]. For instance, in the course of Pol II elongation, both actin and hnRNP associate with the Ser2 phosphorylated CTD Pol II promoting efficient recruitment of the histone acetyltransferases (HATs), which in turn support the unfolding of chromatin and transcription progression [50,51]. Association of nuclear actin with hnRNP has been reported in various species [52–55], and disturbance of this interaction affects the down-regulation of Pol II transcription [56]. Moreover, it has been shown that among the six actin isoforms, specifically β-actin is found in complex with Pol II, hnRNPs and proteins associated with nascent transcripts and these complexes require β-actin in its monomeric (G-actin) form [51]. Noteworthy, a recent report described the presence of endogenous β-actin in the nucleus of the human melanoma A375 cell line. The study further revealed that nuclear β-actin is mainly monomeric and co-localizes with Pol II and hnRNP U, suggesting that nuclear β-actin plays a role in melanoma cells transcription regulation [57]. In this context, it is significant that our confocal imaging showed that AKT co-localizes with active Pol II phosphorylated at Ser2 in the CTD. Moreover, our results suggesting that cofilin is part of the nuclear AKT/β-actin complex also support the notion that AKT might be recruited with β-actin at sites of transcription. Cofilin has been identified as a mediator for the import of actin to the nucleus. Unlike actin, cofilin contains a functional nuclear localization signal (NLS) and monomeric G-actin translocates to the nucleus in complex with cofilin, where cofilin mediates actin transport through association with Importin-9, a nuclear import receptor [60]. Additionally, cofilin has recently been shown to associate with phosphorylated Pol II via interaction with actin, facilitating association of elongating Pol II and actin with active genes [36]. In our study we showed evidence that both cofilin and phosphorylated Pol II co-localize with AKT, suggesting that nuclear AKT/β-actin/cofilin complex may be engaged at Pol II sites. Significant, among putative partners of nuclear AKT identified in our MS/MS analysis we found hnRNP. Although further biochemical examination is required to validate the assembly of nuclear AKT and hnRNP in our cell system, a scenario including nuclear AKT/β-actin/Cofilin in association with hnRNP and Pol II raises attractive questions for understanding the contribution of AKT in the transcriptional apparatus (Figure 8). For example, it has been shown that nuclear AKT associates and phosphorylates the transcriptional co-activator p300. Once phosphorylated, p300 is recruited to the ICAM-1 promoter, leading to the acetylation of histones and association with RNA Pol II, facilitating ICAM-1 gene expression [58]. In this view, it is tempting to speculate that nuclear actin could operate as an interaction platform for nuclear AKT, connecting AKT to transcriptional activators/co-activators via protein–protein interaction to the transcriptional machinery. In a similar fashion, it has been described that the tyrosine kinase receptor ErbB2 translocates to the nucleus and physically associates with nuclear β-actin in breast cancer cells. ErbB-2 enhances the binding of RNA Pol I to rDNA and associates with rDNA concomitantly with β-actin and RNA Pol I, increasing rDNA transcription by RNA Pol I [59].

## Conclusion

The results presented in this report uncovered a hitherto unrecognized molecular coupling of AKT and actin in melanoma cells nuclei and provide insights into the involvement of AKT in the interaction network of nuclear actin. Given the contribution of nuclear actin and actin-binding proteins in the regulation of central nuclear functions, we believe our findings offer a baseline for future studies to explore the role of nuclear AKT activity in the control of actin dynamics in the nucleus as well as of components that operate in concert with nuclear actin, which may shed light on how these interactions contribute to the regulation of key nuclear processes such as transcription and gene expression.

## Supporting information

**Figure S1:** MS/MS spectra from all peptides identified on Table S1.

**Table S1:** The protein and Peptides information identified by MS/MS analysis.

## Abbreviations

ABP: actin-binding protein
CTD: carboxy terminal domain
CREB: cAMP response elemento-binding protein
DSP: dithiobis [succinimidyl propionate]
DSS: succinimidyl suberate
F-actin: filamentous actin (polymer)
G-actin: globular actin (monomer)
HAT: histone acetyltransferase
hnRNP: heterogeneous nuclear ribonucleoprotein
LC-MS: liquid chromatography-mass spectrometry
NLS: nuclear localization sequence
Pol II: RNA polymerase II
