# Supplementary material for: AKT/protein kinase B associates with β-actin in the nucleus of melanoma cells
Source: Biosci Rep. 2019 Jan 30;39(1):BSR20181312. doi: 10.1042/BSR20181312 (PMC6356016; doi:10.1042/BSR20181312)
Supplement: Supplementary file 1 [file bsr20181312_Supp1.pdf]

Supplementary Material

Table S1 The protein and Peptides information identified by MS/MS analysis.

Table S1. The protein and Peptides information identified by MS/MS.

Cell line	Treatment	Protein Group	Protein ID	Protein Accession	Peptide	Unique	-10lgP	Mass	Length	ppm	m/z	z	RT	Scan	#Spec	Start	End	PTM
A2058	unstimulated	3	119661	P60709|ACTB_HUMAN (β-actin)	K.SYELPDGQVITIGNER.F	N	98,36	1789,88	16	0,5	1790,9	1	-	F27:80521	19	239	254	
					R.VAPEEHPVLLTEAPLNPK.A	N	84,57	1953,06	18	1,6	1954,1	1	-	F26:66857	16	96	113	
					R.GYSFTTTAER.E	N	77,98	1131,52	10	2,6	1132,5	1	-	F27:75738	33	197	206	
					K.QEYDESGPSIVHR.K	N	76,57	1515,7	13	0,8	1516,7	1	-	F7:17553	11	360	372	
					K.AGFAGDDAPR.A	N	71,26	975,441	10	1,8	976,45	1	-	F27:74647	17	19	28	
					R.AVFPSIVGRPR.H	N	69,02	1197,7	11	1,5	1198,7	1	-	F27:77396	14	29	39	
					K.DSYVGDEAQSKR.G	N	67,08	1353,62	12	3,3	1354,6	1	-	F27:73773	2	51	62	
					K.EITALAPSTM(+15.99)K.I	N	56,31	1176,61	11	1,2	1177,6	1	-	F28:87183	12	316	326	Oxidation (M)
					V.FPSIVGRPR.H	N	51,48	1027,59	9	3,7	1028,6	1	-	F27:77387	12	31	39	
					R.DLTDYLM(+15.99)K.I	N	45,81	1013,47	8	1,6	1014,5	1	-	F27:79586	9	184	191	Oxidation (M)
					F.PSIVGRPR.H	N	41,49	880,524	8	1,2	881,53	1	-	F27:77392	6	32	39	
					R.AVFPSIVGR.P	N	38,14	944,544	9	1	945,55	1	-	F27:79072	7	29	37	
		79	119973	P22626-2 ROA2_HUMAN (Heterogeneous nuclear ribonucleoproteins A2/B1)	R.GGNFGFGDSR.G	Y	40,18	1012,44	10	-0,6	1013,4	1	-	F1:348	8	192	201	
					R.GGGGNFGPGPGSNFR.G	Y	35,07	1376,62	15	2,3	1377,6	1	-	F8:22395	7	202	216	
					K.IDTIEIITDR.Q	Y	31,45	1187,64	10	-1	1188,6	1	-	F1:751	5	126	135	
A2058	serum stimulated	4	119659	P63261 ACTG_HUMAN (γ-actin)	R.VAPEEHPVLLTEAPLNPK.A	N	84,57	1953,06	18	1,6	1954,1	1	-	F26:66857	16	96	113	
					R.GYSFTTTAER.E	N	77,98	1131,52	10	2,6	1132,5	1	-	F27:75738	33	197	206	
					K.AGFAGDDAPR.A	N	71,26	975,441	10	1,8	976,45	1	-	F27:74647	17	19	28	
					R.AVFPSIVGRPR.H	N	69,02	1197,7	11	1,5	1198,7	1	-	F27:77396	14	29	39	
					K.DSYVGDEAQSKR.G	N	67,08	1353,62	12	3,3	1354,6	1	-	F27:73773	2	51	62	
					K.EITALAPSTMK.I	N	58,31	1160,61	11	2,5	1161,6	1	-	F27:77306	5	316	326	
					R.DLTDYLMK.I	N	46,12	997,479	8	-1,8	998,48	1	-	F27:82396	6	184	191	
		14	119776	Q562R1 ACTBL_HUMAN (β-actin-like protein 2)	R.SYELPDGQVITIGNER.F	N	98,36	1789,88	16	0,5	1790,9	1	-	F27:80521	19	240	255	
					V.PIYEGYALPHAILR.L	N	72,99	1611,88	14	0,9	1612,9	1	-	F27:80647	3	165	178	
					R.TTGIVMDSGD.G	N	60,34	994,428	10	0,1	995,44	1	-	F27:77623	1	149	158	
					I.YEGYALPHAILR.L	N	53,11	1401,74	12	1	1402,7	1	-	F26:67541	2	167	178	
					R.DLTDYLMK.I	N	46,12	997,479	8	-1,8	998,48	1	-	F27:82396	6	185	192	
					K.IKIIAPPERK.Y	N	45,56	1163,74	10	0,8	1164,7	1	-	F26:62401	1	328	337	
					V.GDEAQSK.R	N	34,52	733,324	7	-0,8	734,33	1	-	F26:62047	4	56	62	
					D.GQVITIGNER.F	N	31,46	1085,58	10	3,4	1086,6	1	-	F27:75874	2	246	255	
					R.VAPDEHPILLTEAPLNPK.I	Y	25,35	1953,06	18	-0,2	1954,1	1	-	F5:11617	1	97	114	
					E.KIWYHTFYNELR.V	Y	24,21	1668,84	12	1,6	1669,9	1	-	F27:82758	1	85	96	
		1	119658	P08670 VIME_HUMAN (vimentin)	R.DGQVINETSQHHDDL.E	Y	81,15	1835,79	16	0,9	1836,8	1	-	F27:75635	21	451	466	
					R.EYQDLLNVK.M	N	72,59	1120,58	9	-1,8	1121,6	1	-	F26:66771	18	382	390	
					K.FADLSEAANR.N	N	61,04	1092,52	10	-0,4	1093,5	1	-	F6:14147	15	295	304	
					R.QDVDNASLAR.L	N	56,89	1087,53	10	-1	1088,5	1	-	F7:17283	11	208	217	
					K.VELQELNDR.F	N	54,22	1114,56	9	0,9	1115,6	1	-	F27:76106	15	105	113	
					R.DNLAEDIMR.L	N	54,09	1075,5	9	2	1076,5	1	-	F28:90052	13	176	184	
					R.LGDLYEEEMRELR.R	N	43,38	1651,79	13	0	1652,8	1	-	F26:67323	3	146	158	
					K.LLEGEESR.I	N	40,34	931,461	8	4	932,47	1	-	F27:74337	12	403	410	

Supplementary Material

Figure S1 MS/MS spectra from all peptides identified on Table S1.

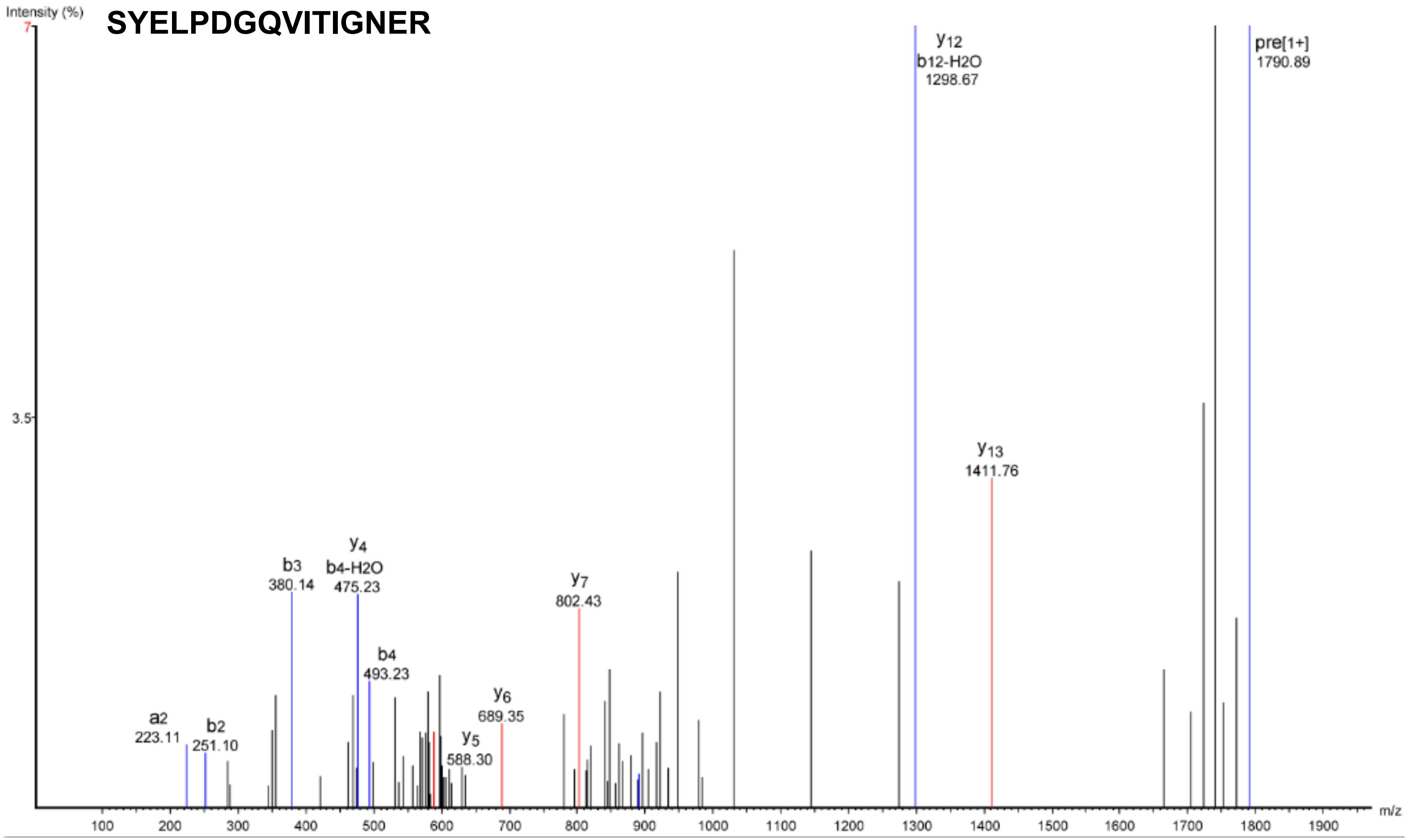

MS/MS spectrum of the peptide SYELPDGQVITIGNER identified from actin (P60709). Deisotoped peptide ion m/z: 1790.89^{+1}.

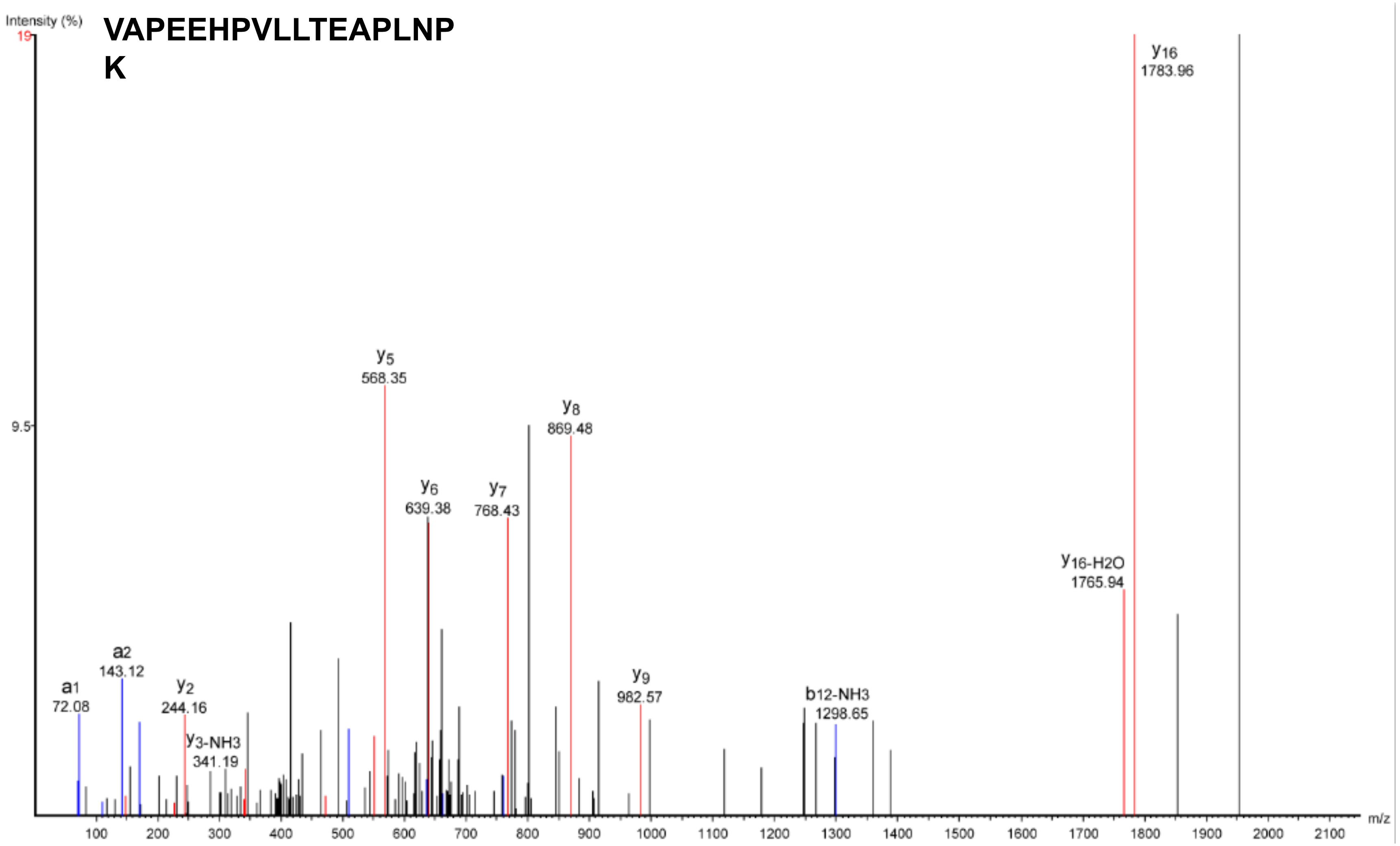

MS/MS spectrum of the peptide VAPEEHPVLLTEAPLNPK identified from actin (P60709). Deisotoped peptide ion m/z: 1954.06^{+1}.

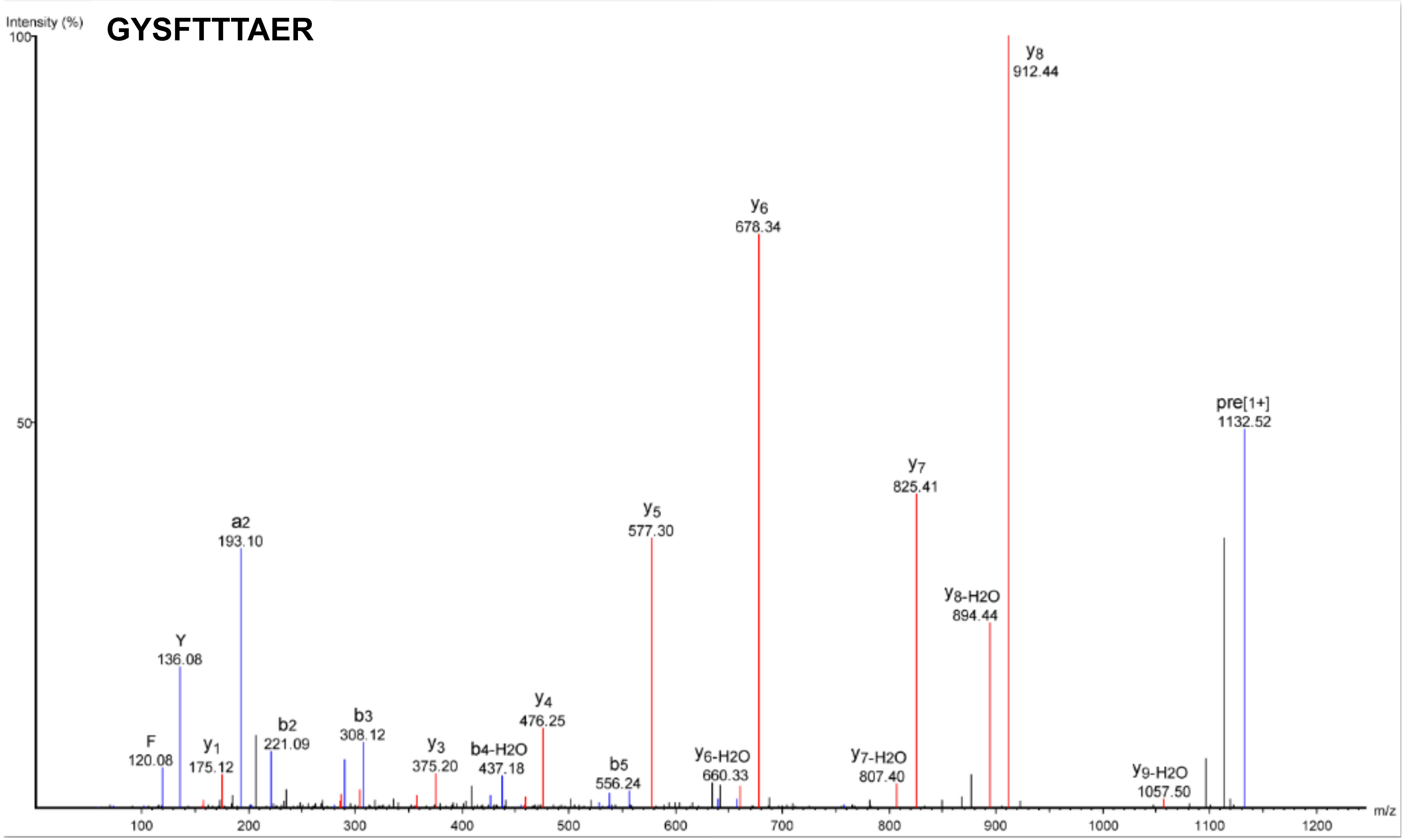

MS/MS spectrum of the peptide GYSFTTTAER identified from actin (P60709). Deisotoped peptide ion m/z: 1132.52^{+1}.

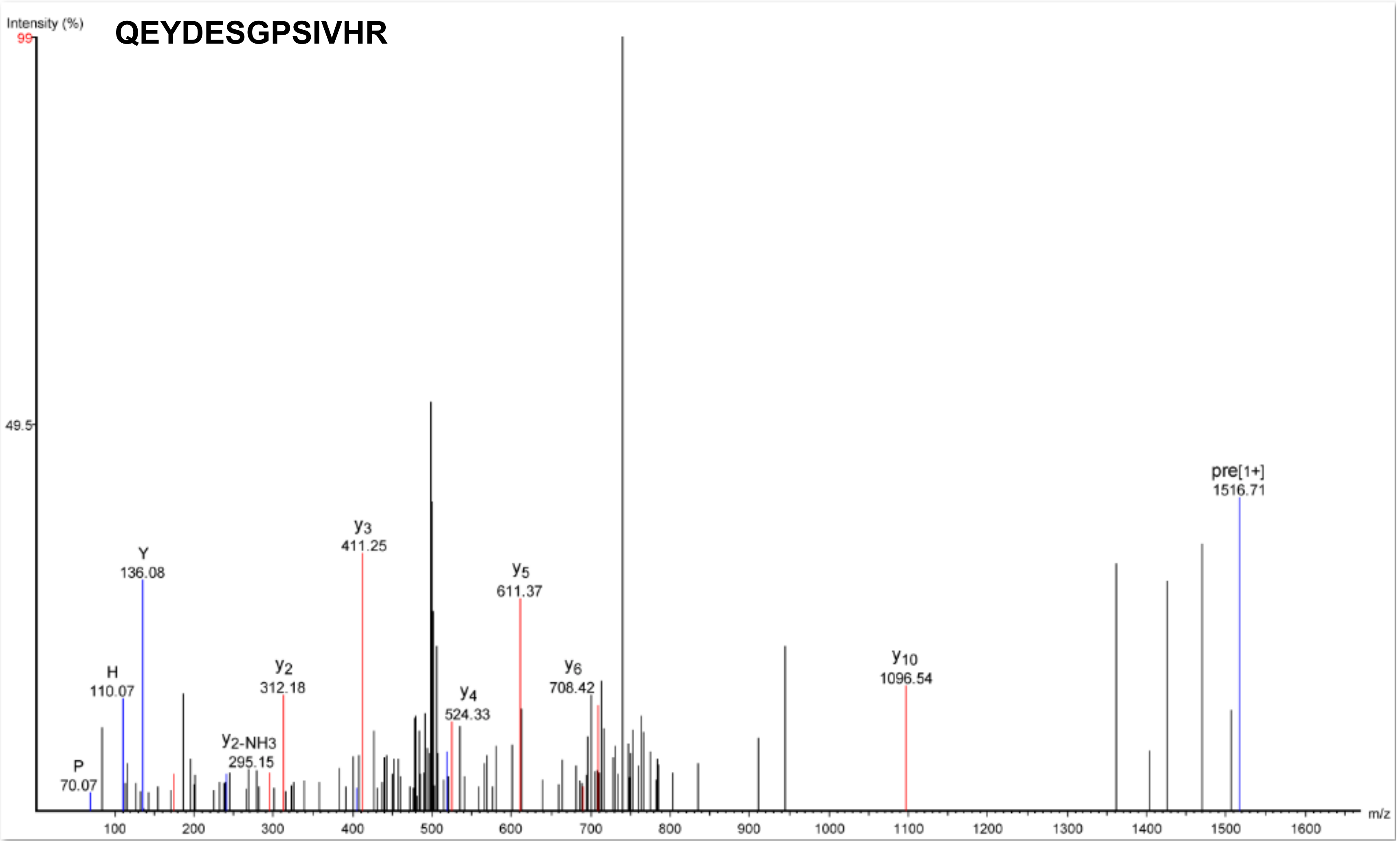

MS/MS spectrum of the peptide QEYDESGPSIVHR identified from actin (P60709). Deisotoped peptide ion m/z: 1516.71^{+1}.

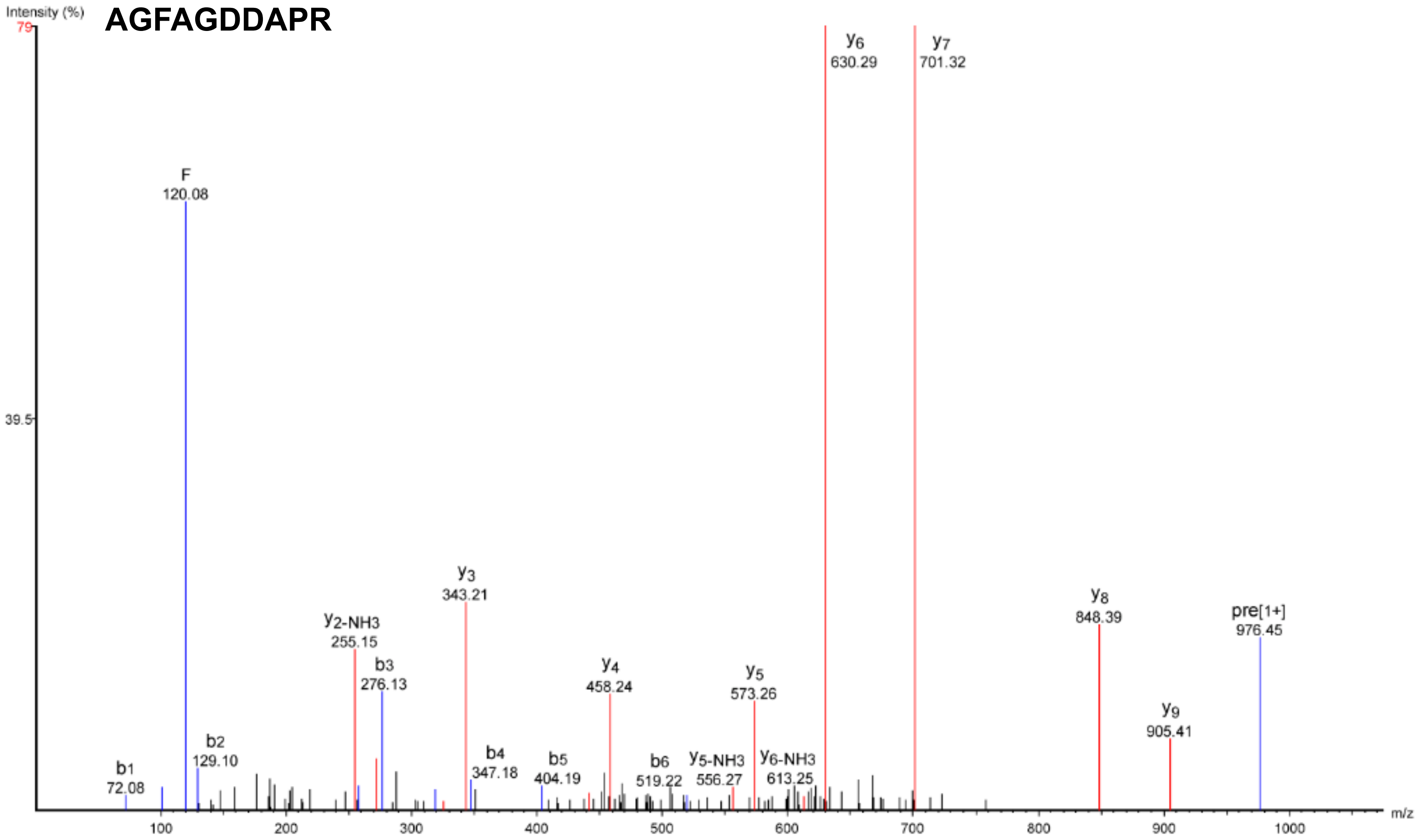

MS/MS spectrum of the peptide AGFAGDDAPR identified from actin (P60709). Deisotoped peptide ion m/z: 976.45^{+1}.

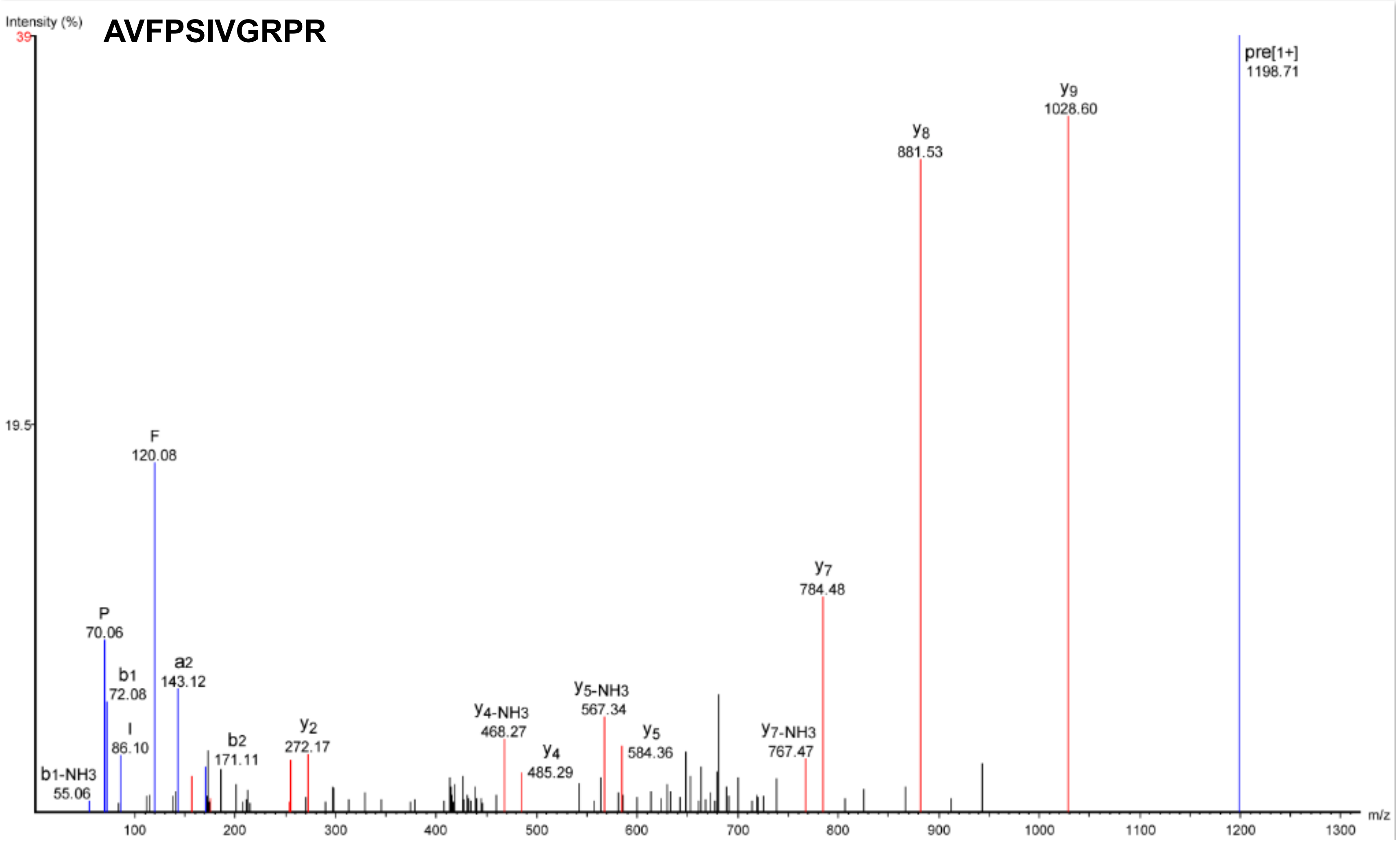

MS/MS spectrum of the peptide AVFPSIVGRPR identified from actin (P60709). Deisotoped peptide ion m/z: 1198.71[+1].

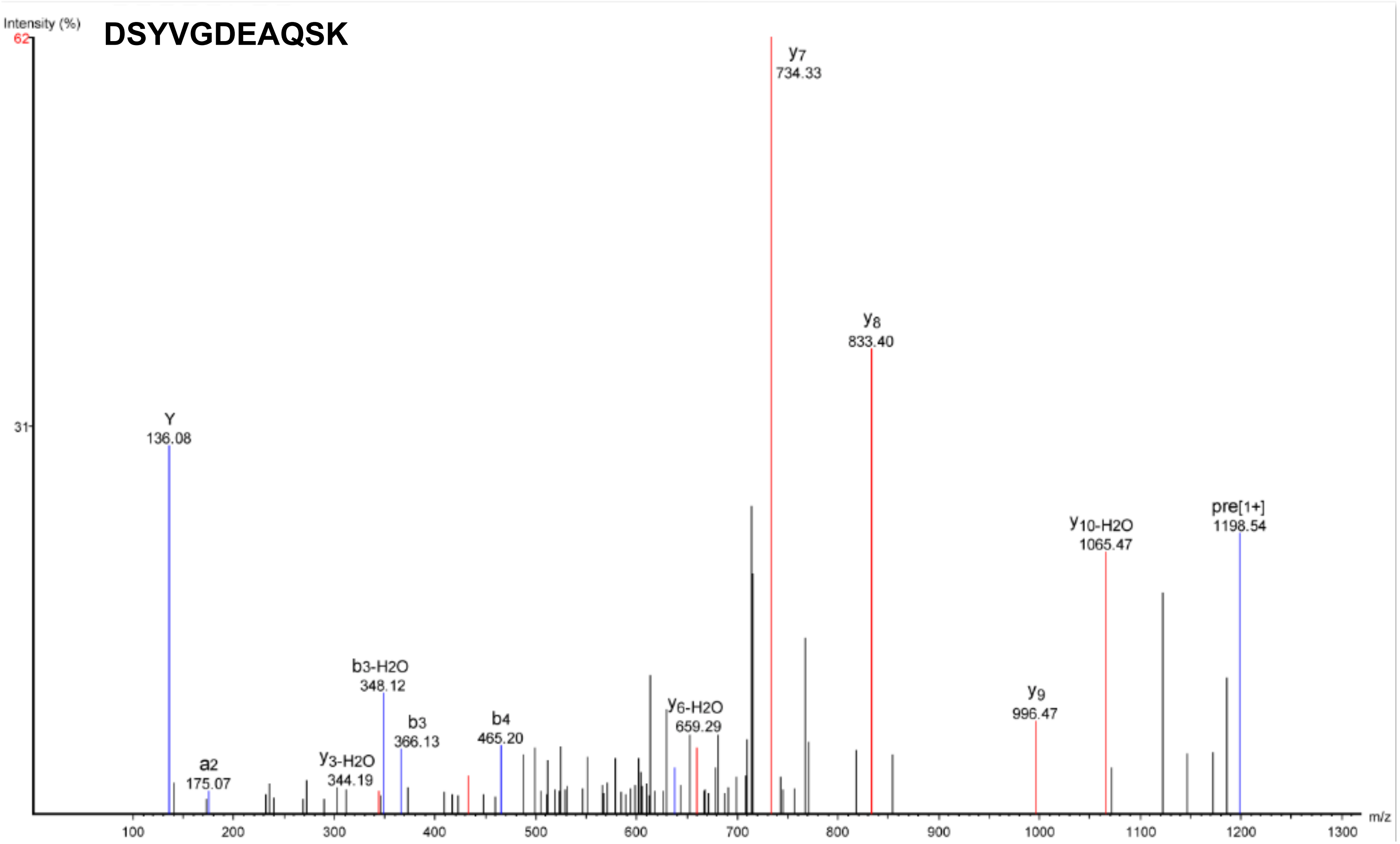

MS/MS spectrum of the peptide DSYVGDEAQSK identified from actin (P60709). Deisotoped peptide ion m/z: 1198.54^{+1}.

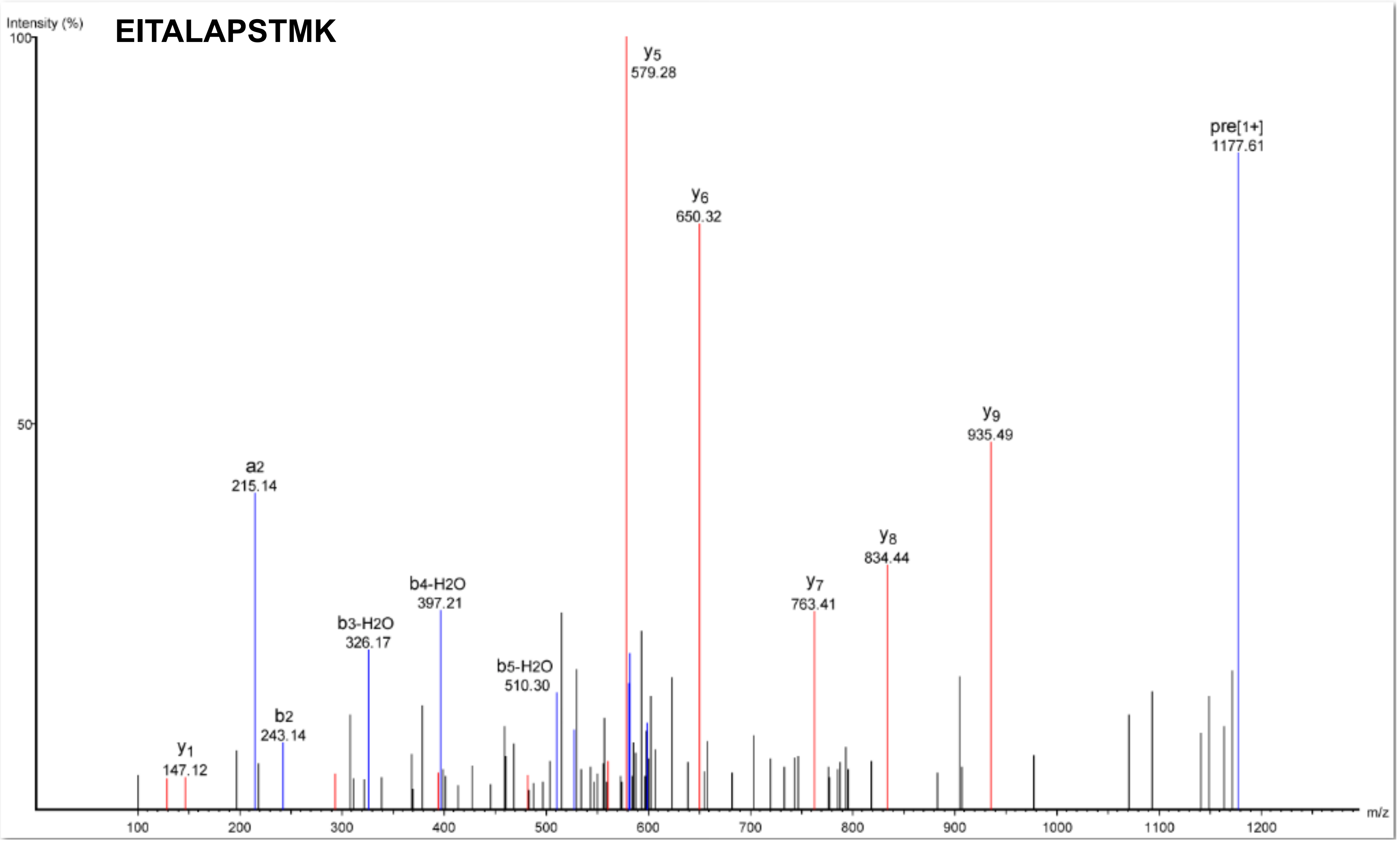

MS/MS spectrum of the peptide EITALAPSTMK identified from actin (P60709). Deisotoped peptide ion m/z: 1177.61^{+1}.

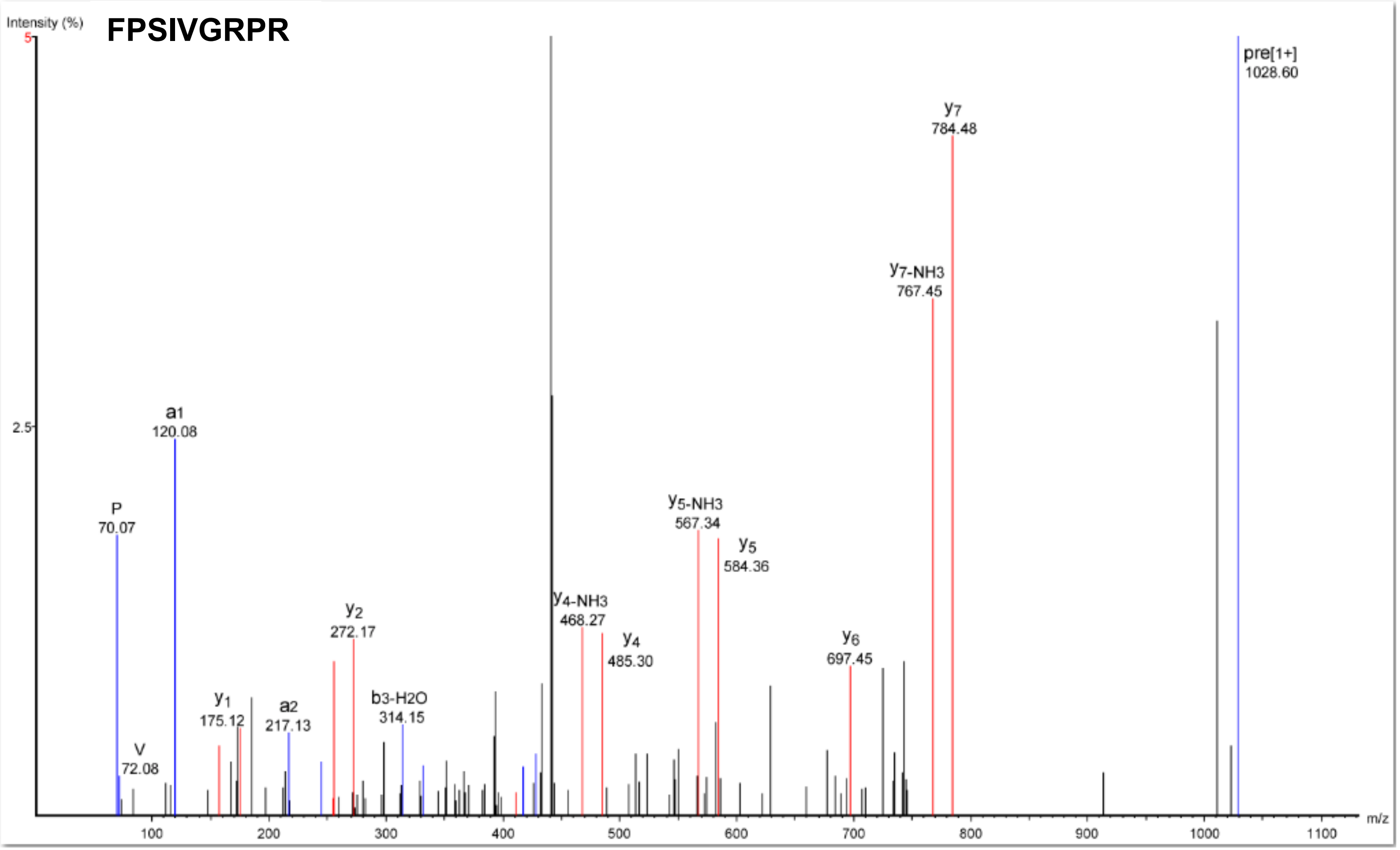

MS/MS spectrum of the peptide FPSIVGRPR identified from actin (P60709). Deisotoped peptide ion m/z: 1028.60^{+1}.

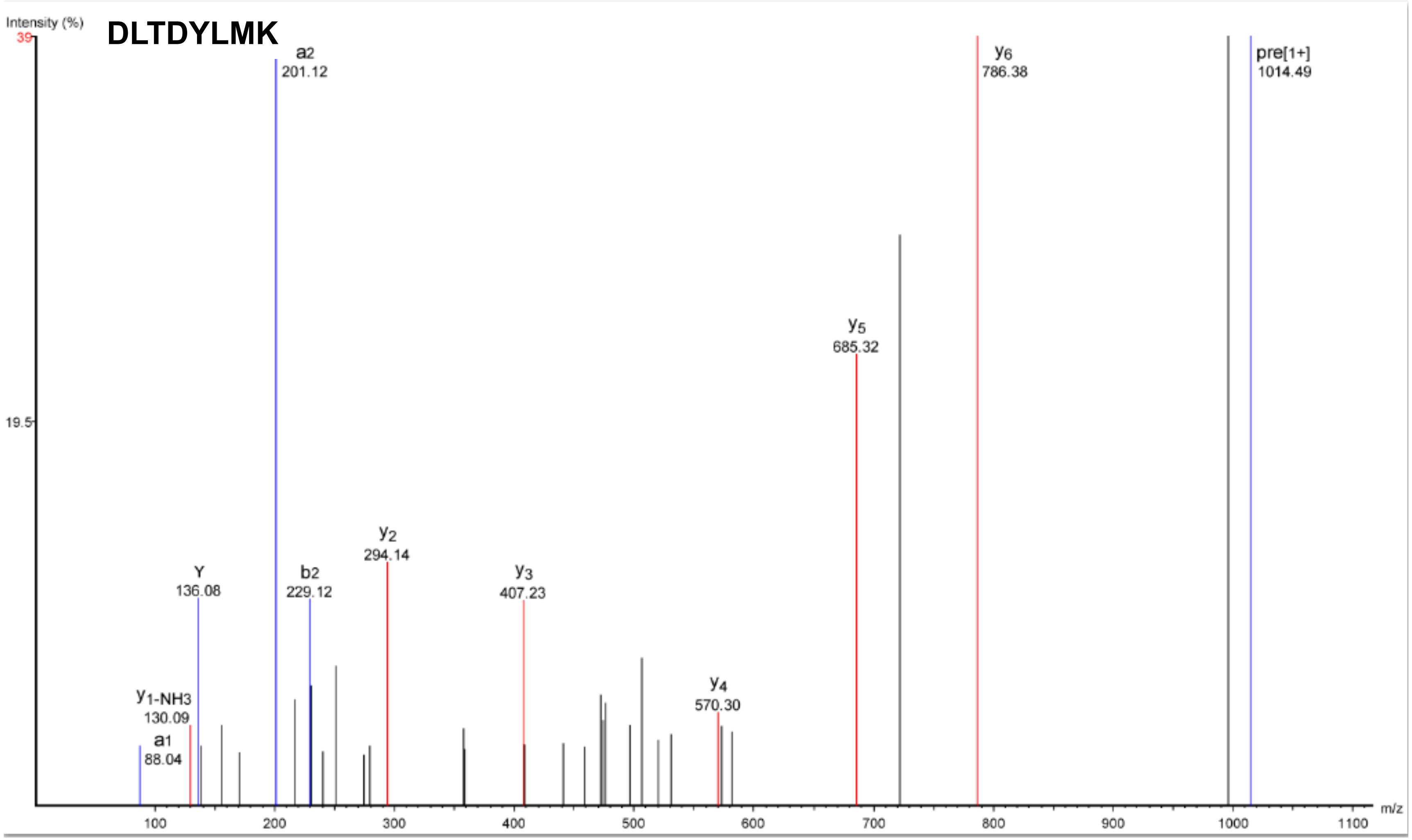

MS/MS spectrum of the peptide DLTDYLMK identified from actin (P60709). Deisotoped peptide ion m/z: 1014.49^{+1}.

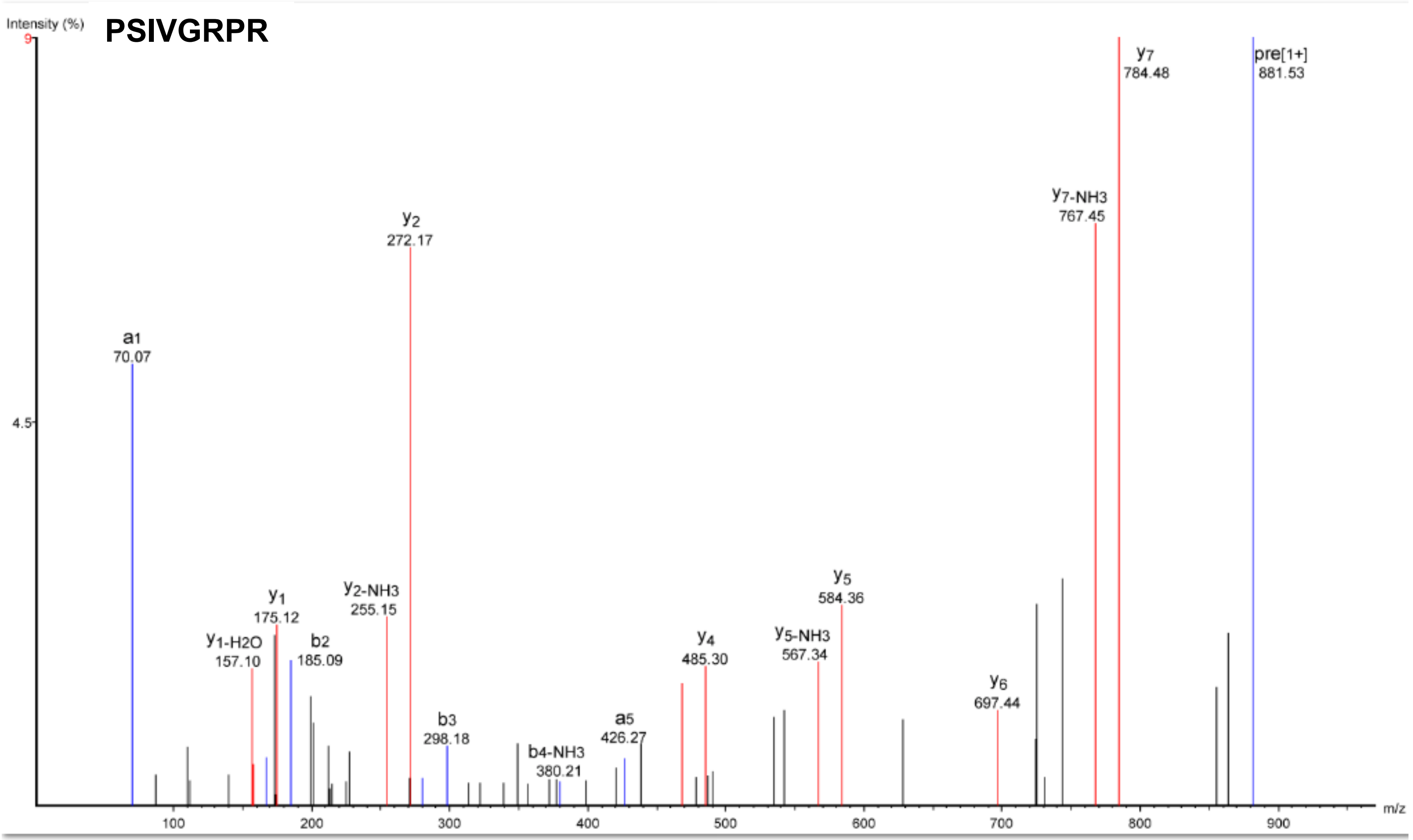

MS/MS spectrum of the peptide PSIVGRPR identified from actin (P60709). Deisotoped peptide ion m/z: 881.53^{+1}.

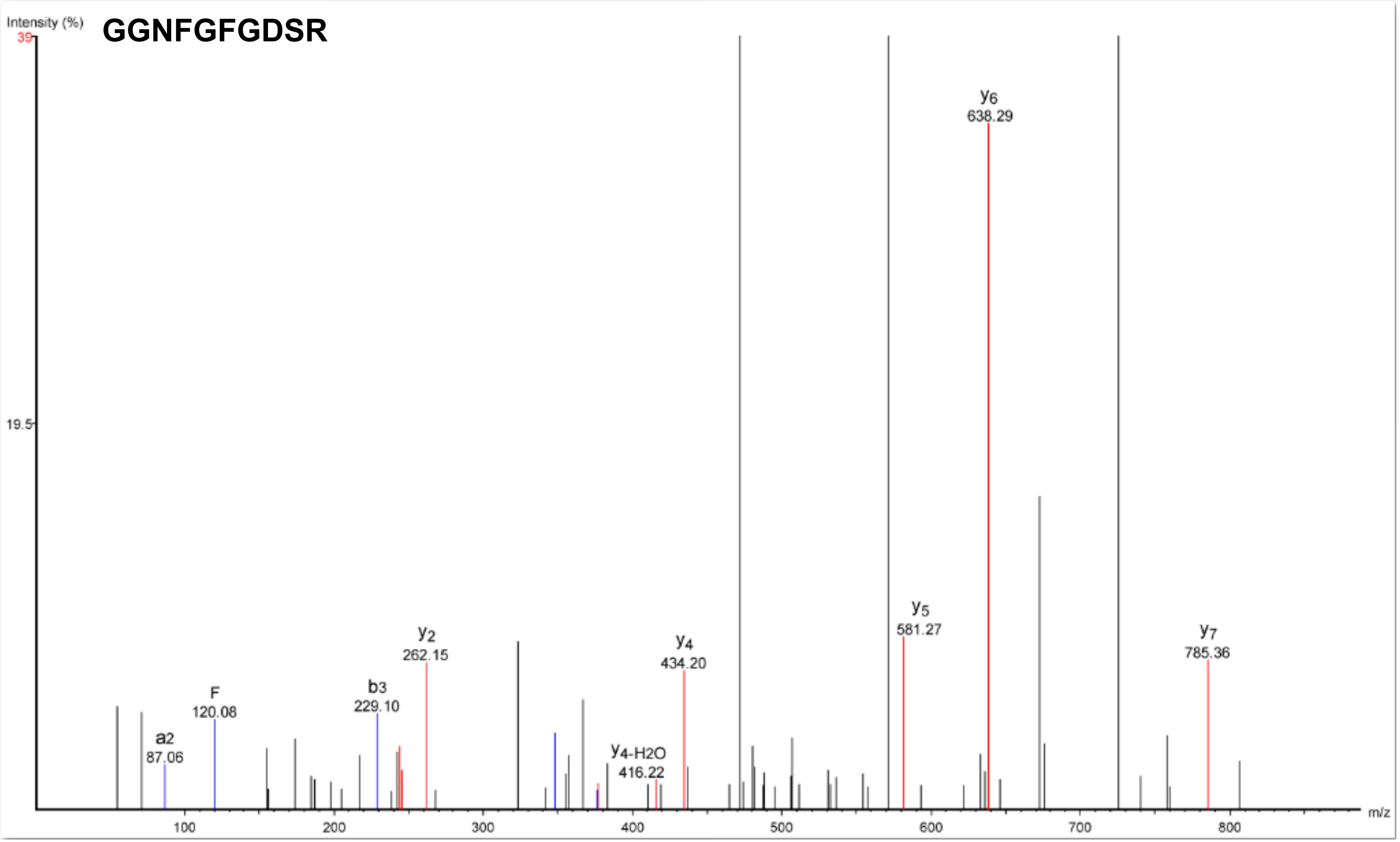

MS/MS spectrum of the peptide GGNFGFGDSR identified from heterogeneous nuclear ribonucleoprotein (P22626). Deisotoped peptide ion m/z: 1013.44^{+1}

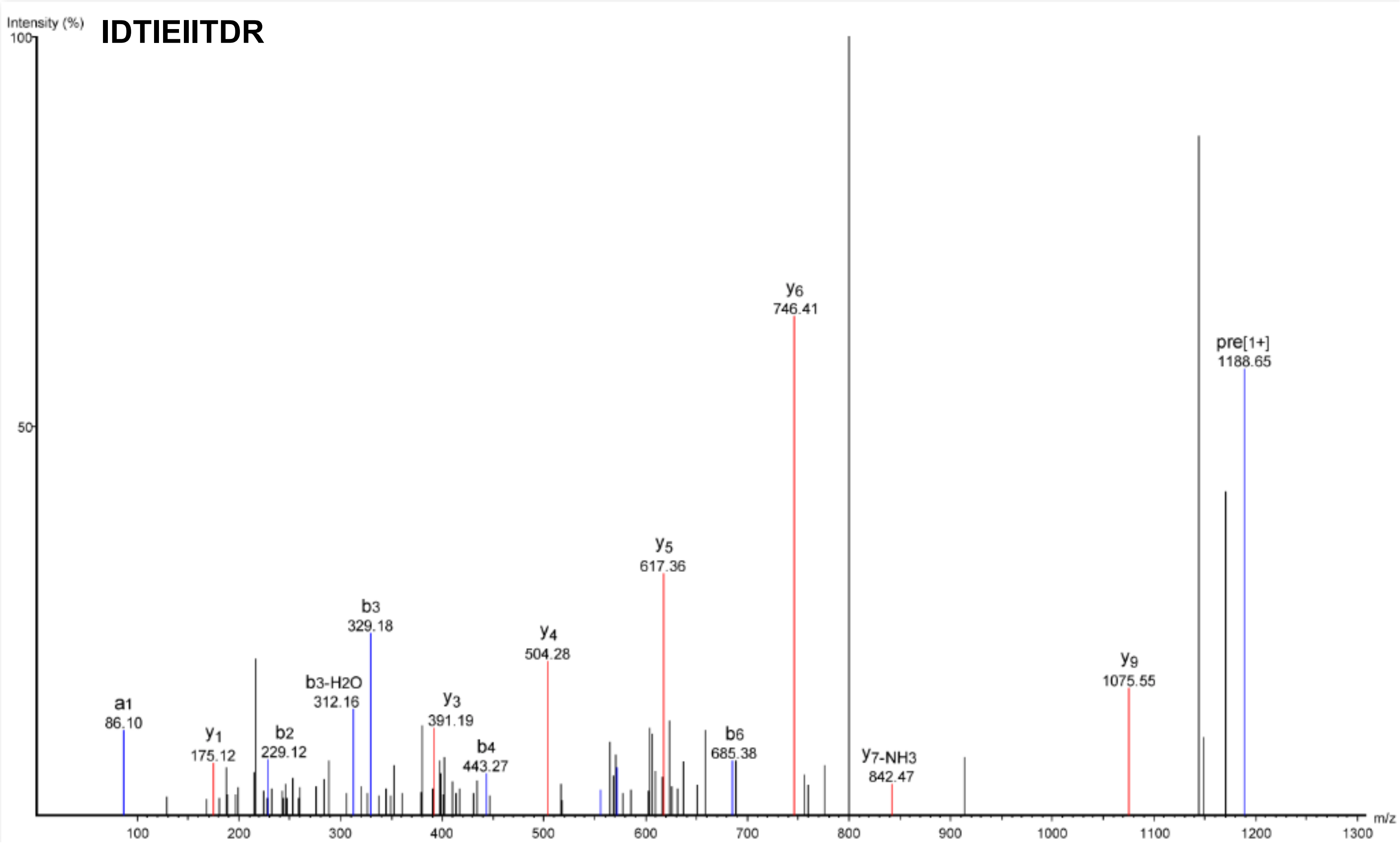

MS/MS spectrum of the peptide IDTIEIITDR identified from heterogeneous nuclear ribonucleoprotein (P22626). Deisotoped peptide ion m/z: 1188.65^{+1}.

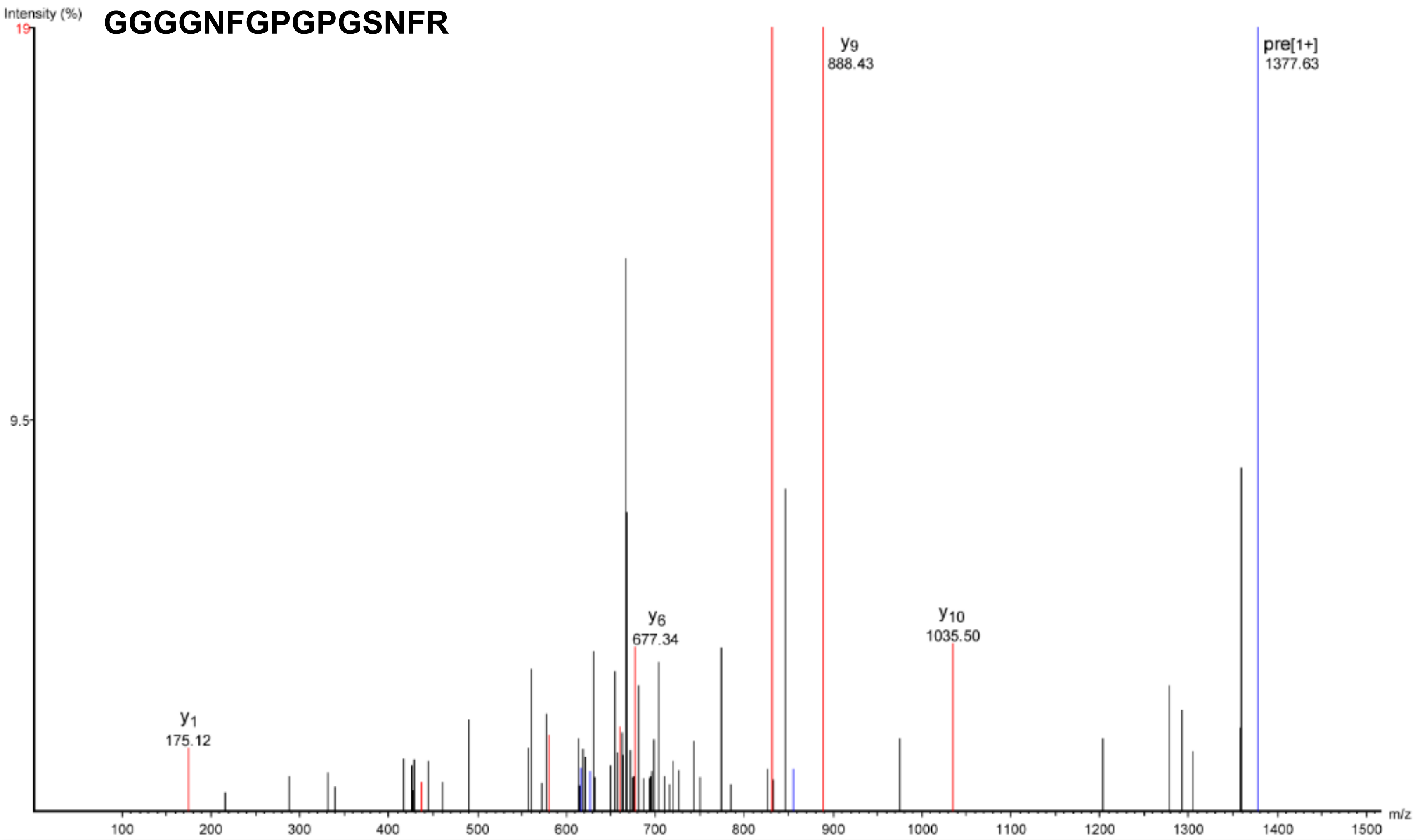

MS/MS spectrum of the peptide GGGGNFGPGPGSNFR identified from heterogeneous nuclear ribonucleoprotein (P22626). Deisotoped peptide ion m/z: 1377.63^{+1}.

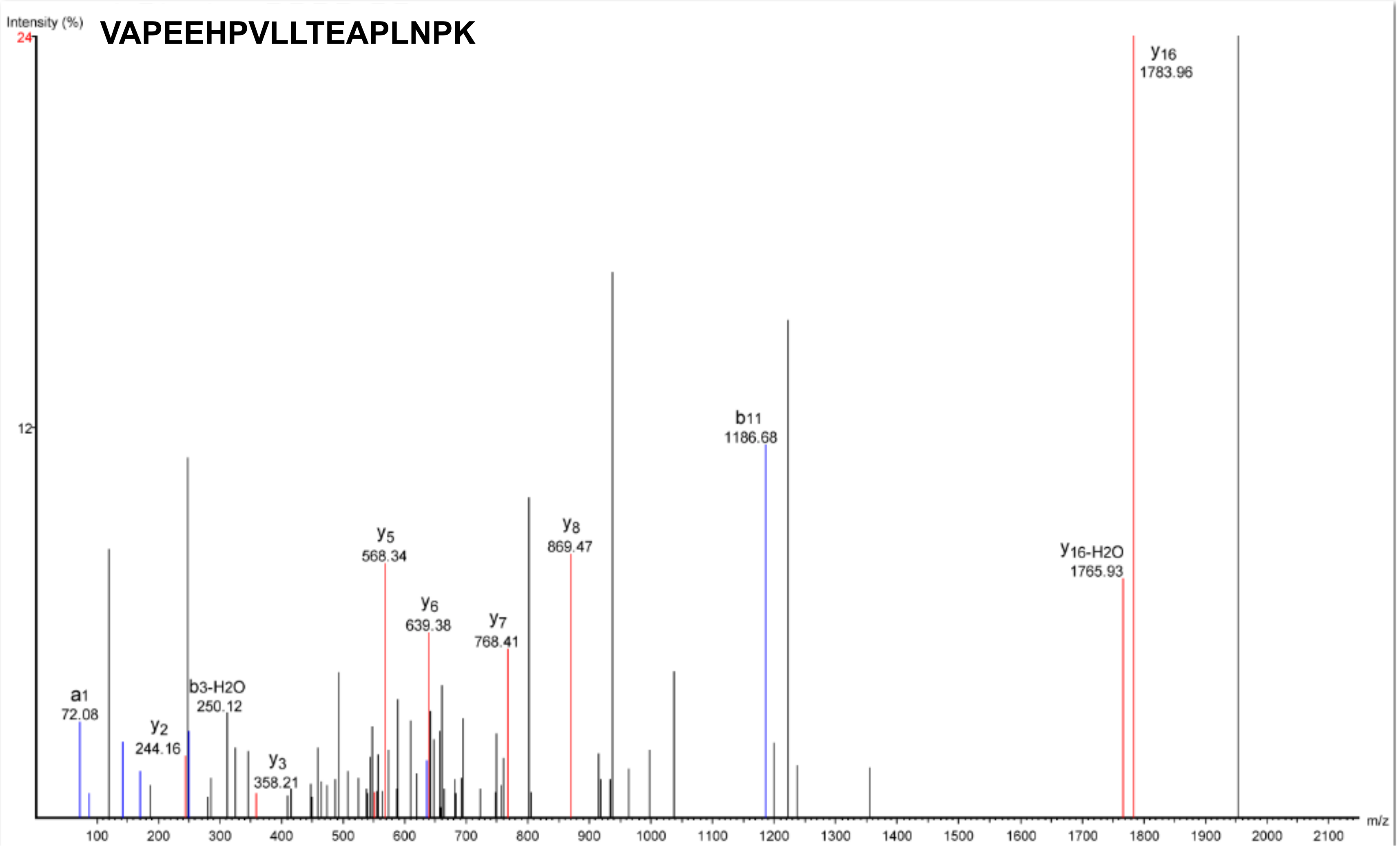

MS/MS spectrum of the peptide VAPEEHPVLLTEAPLNPK identified from actin (P63261). Deisotoped peptide ion m/z: 1954.06^{+1}.

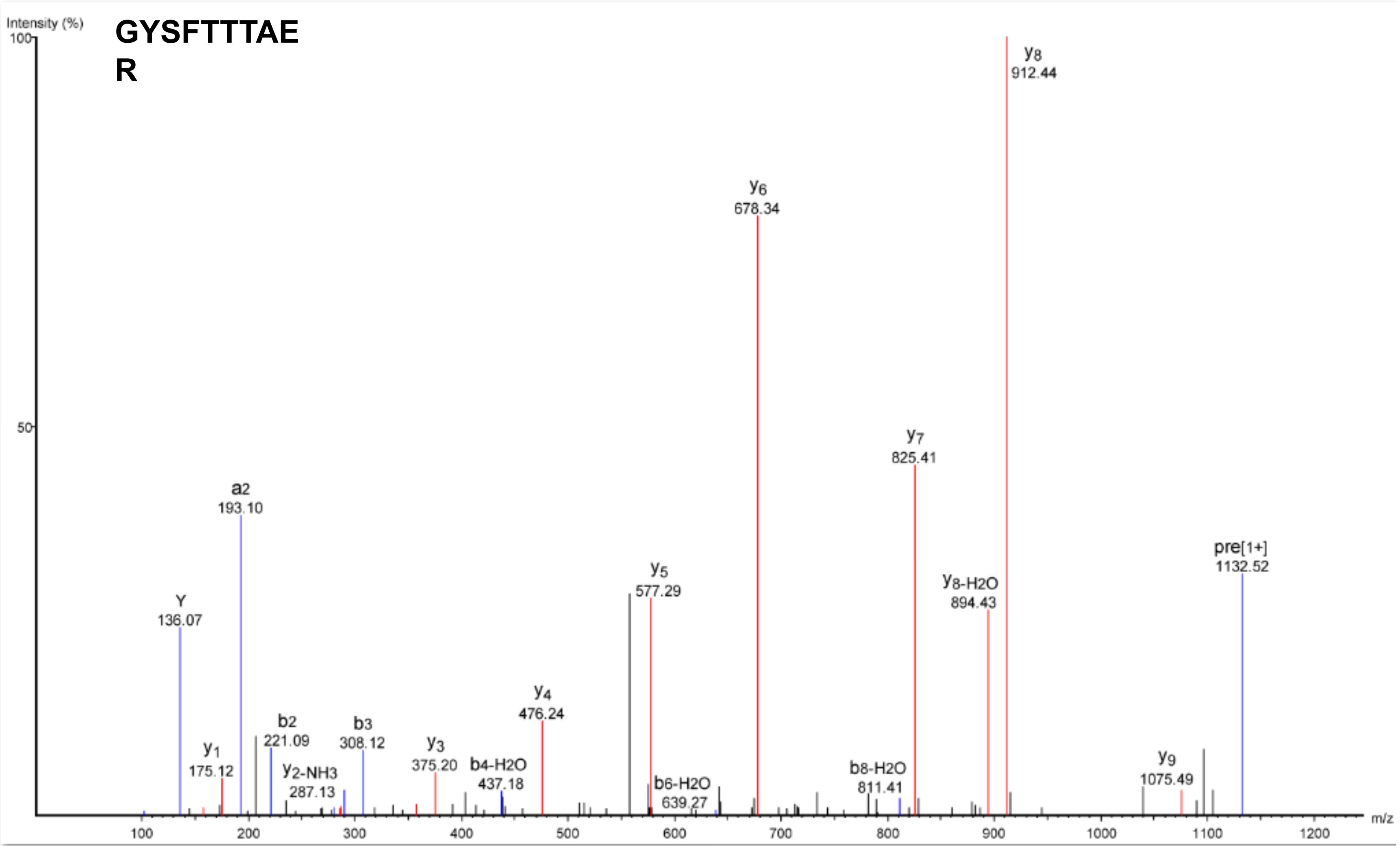

MS/MS spectrum of the peptide GYSFTTTAER identified from actin (P63261). Deisotoped peptide ion m/z: 1132.52^{+1}.

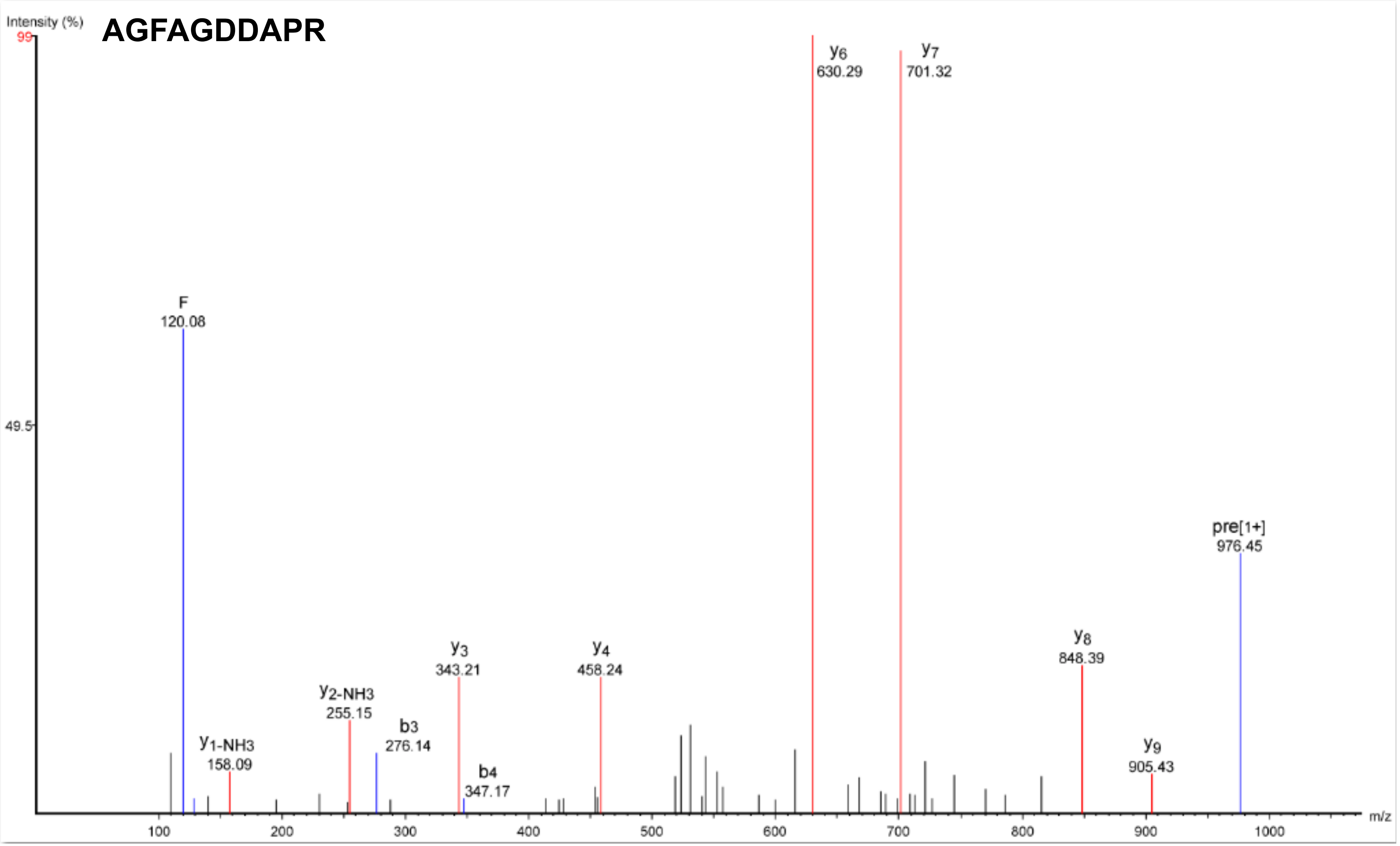

MS/MS spectrum of the peptide AGFAGDDAPR identified from actin (P63261). Deisotoped peptide ion m/z: 976.45^{+1}.

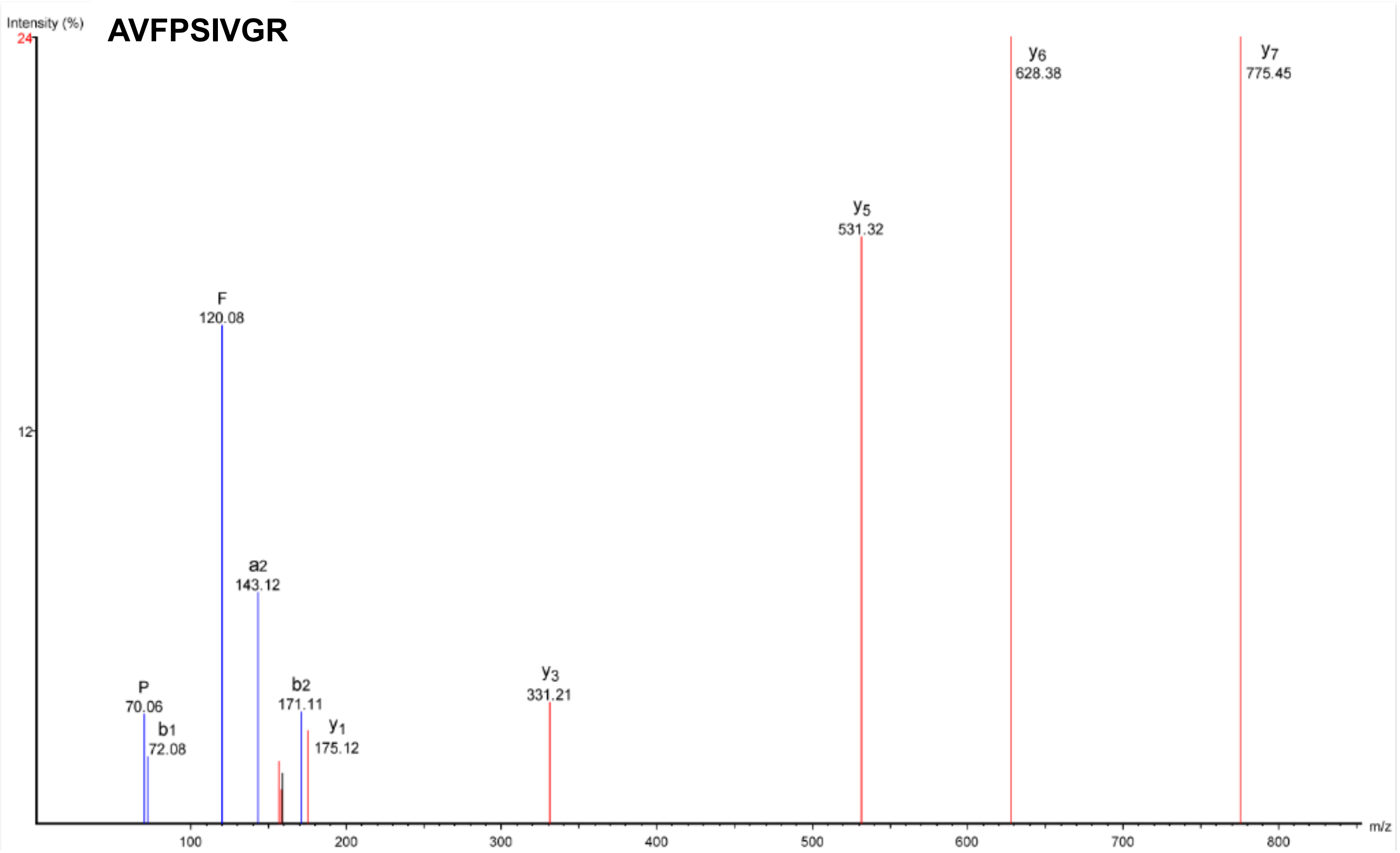

MS/MS spectrum of the peptide AVFPSIVGR identified from actin (P63261). Deisotoped peptide ion m/z: 945.55^{+1}.

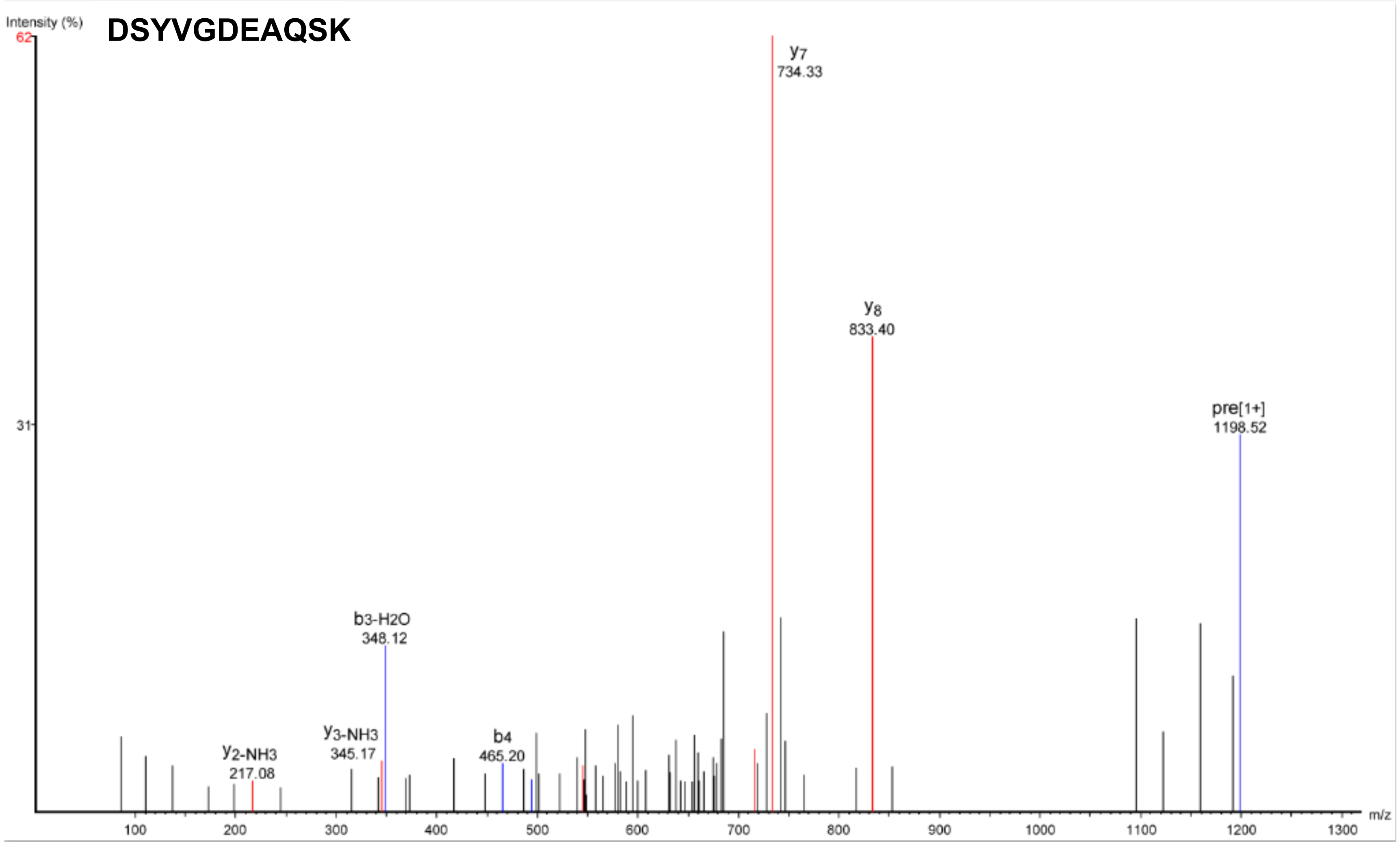

MS/MS spectrum of the peptide DSYVGDEAQSK identified from actin (P63261). Deisotoped peptide ion m/z: 1198.52^{+1}.

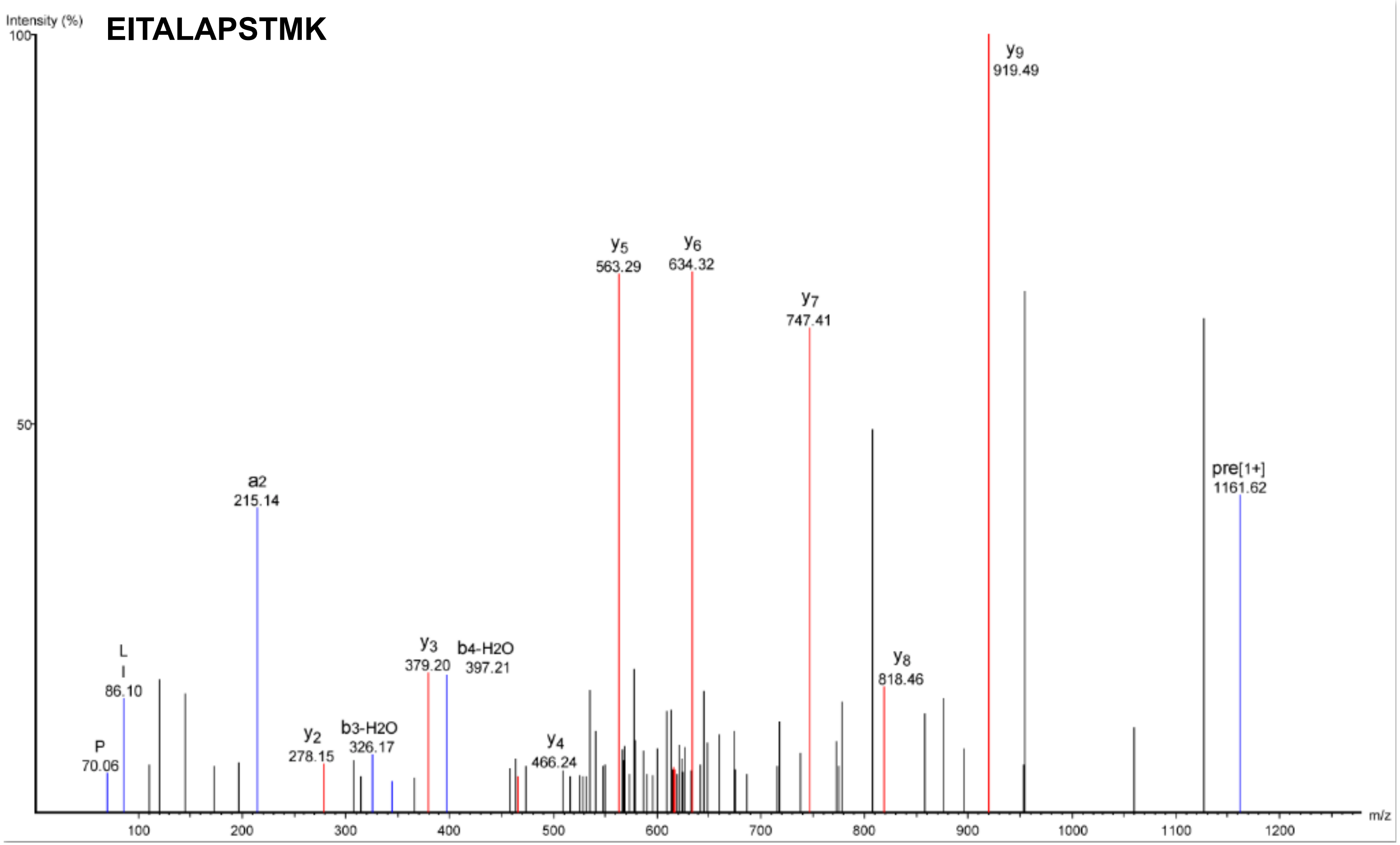

MS/MS spectrum of the peptide EITALAPSTMK identified from actin (P63261). Deisotoped peptide ion m/z: 1161.62^{+1}.

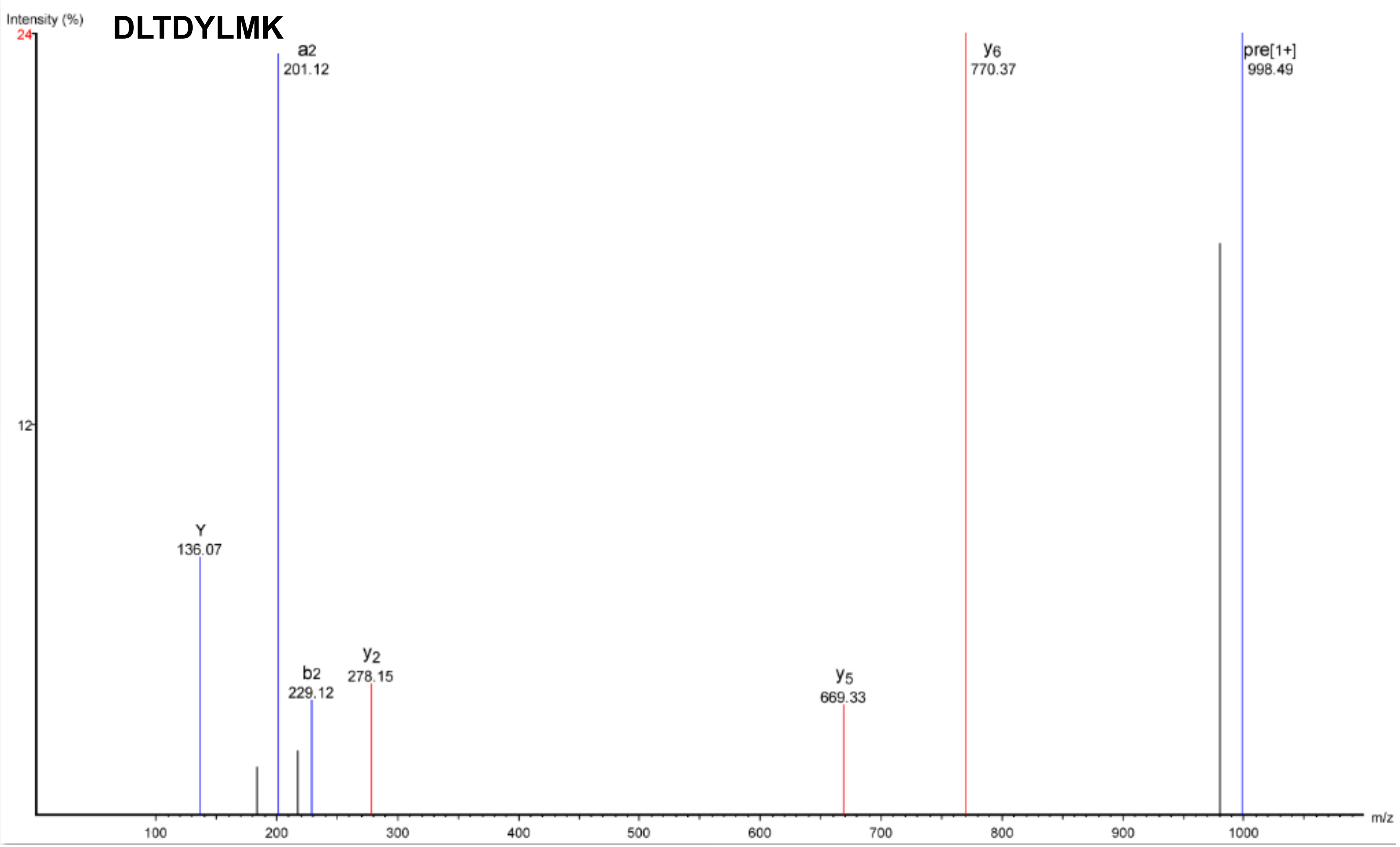

MS/MS spectrum of the peptide DLTDYLMK identified from actin (P63261). Deisotoped peptide ion m/z: 998.49^{+1}.

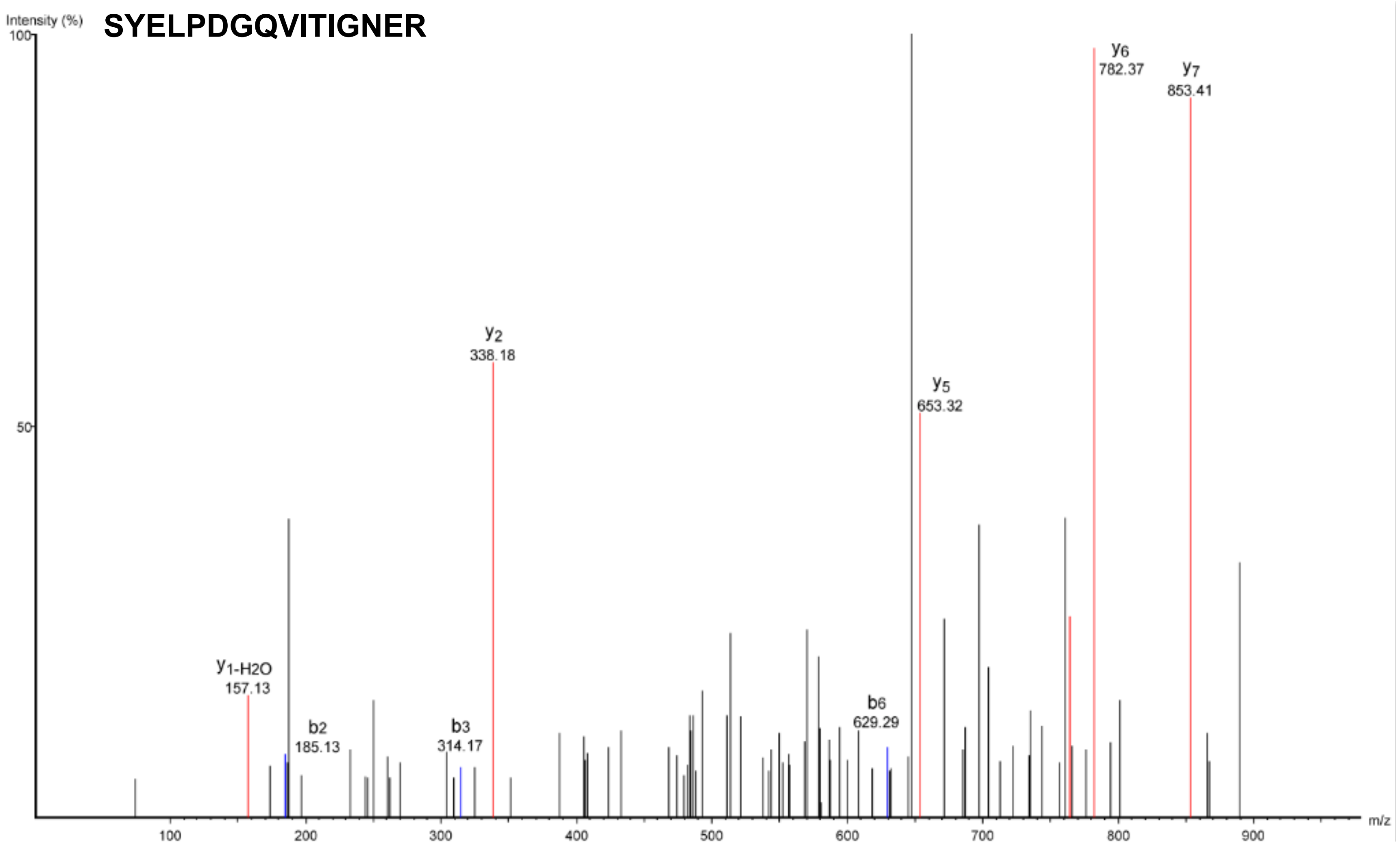

Figure. MS/MS spectrum of the peptide SYELPDGQVITIGNER identified from beta-actin-like protein (Q562R1). Deisotoped peptide ion m/z: 966.48^{+1}.

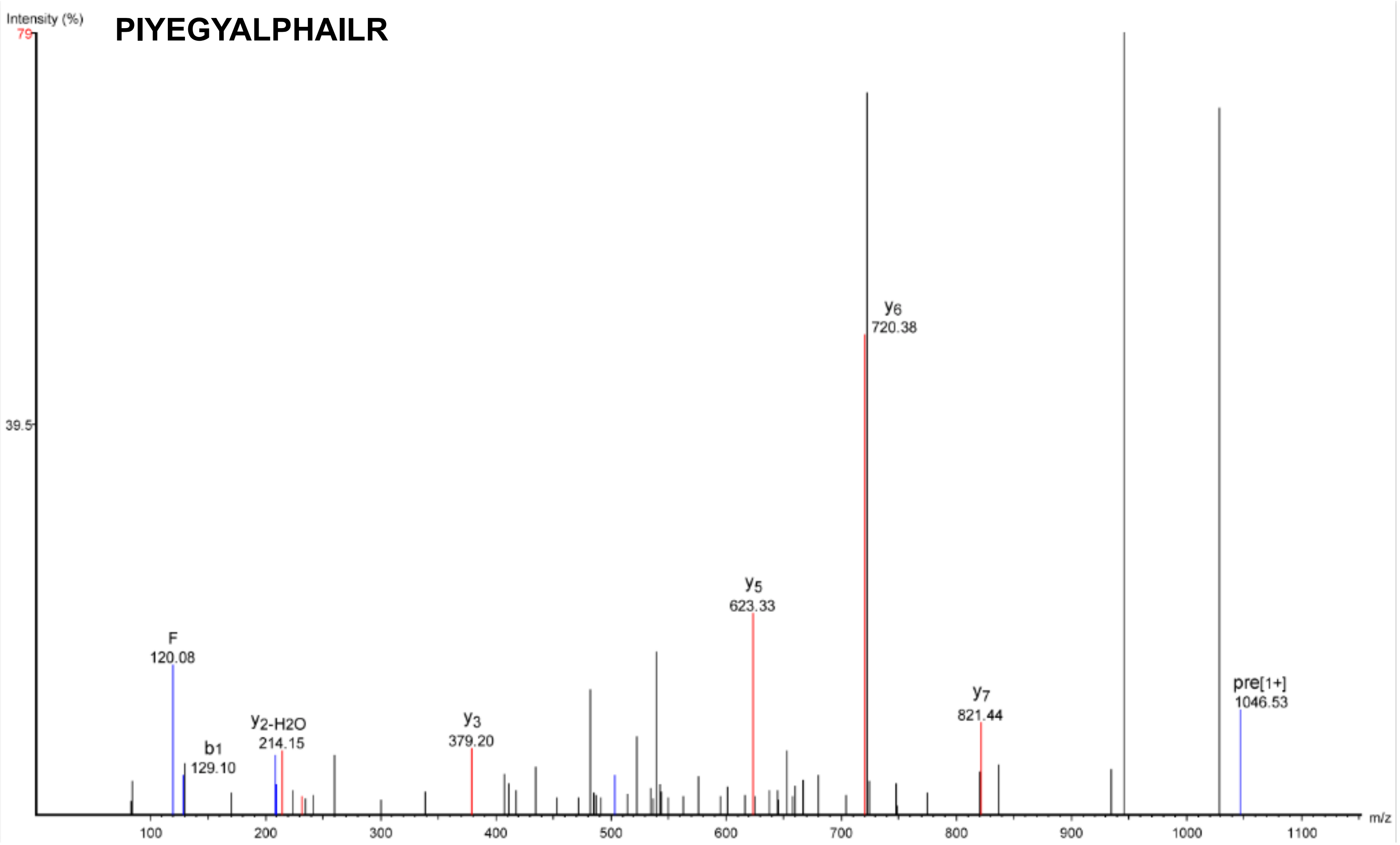

MS/MS spectrum of the peptide PIYEGYALPHAILR identified from beta-actin-like protein (Q562R1). Deisotoped peptide ion m/z: 1046.53^{+1}.

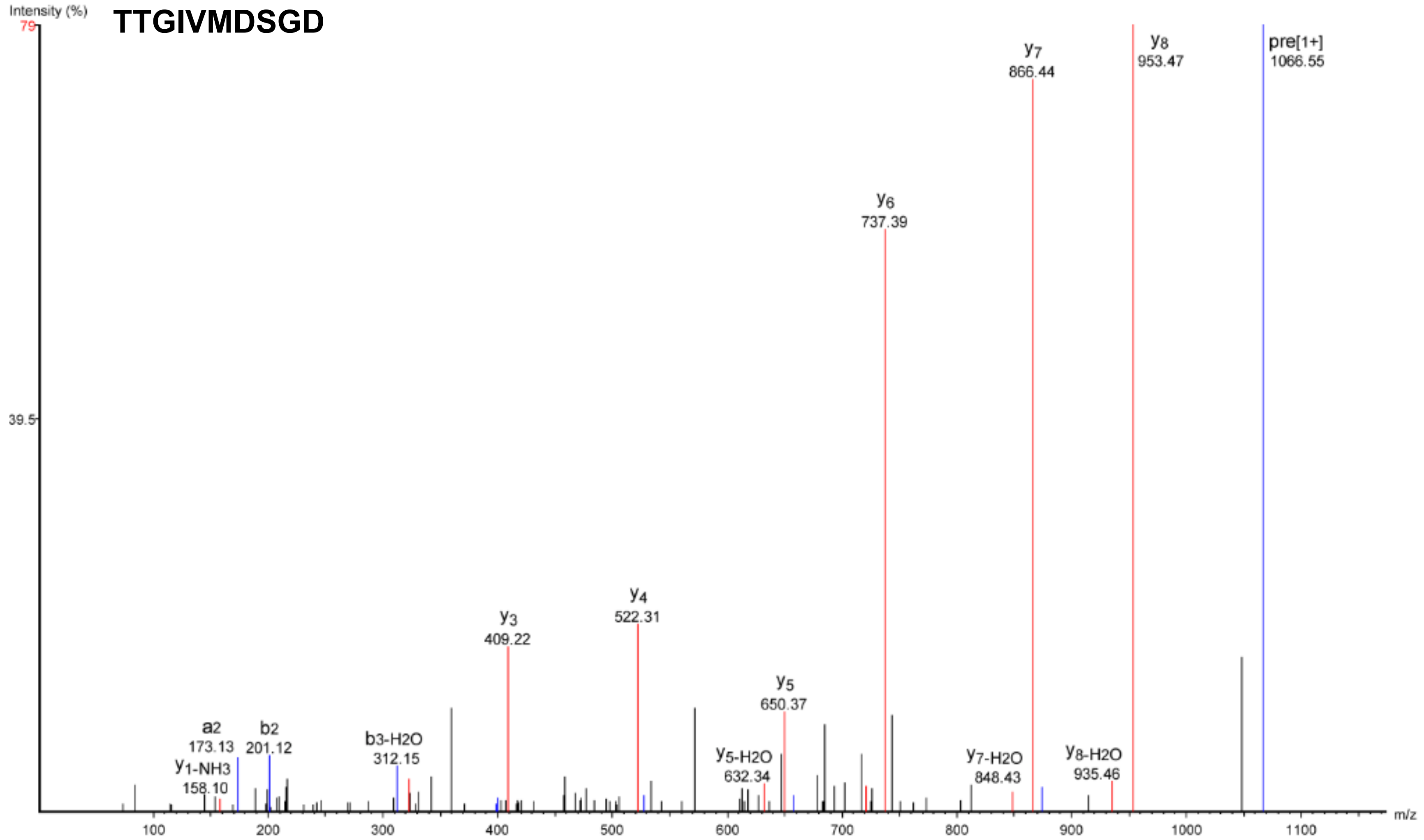

MS/MS spectrum of the peptide TTGIVMDSGD identified from beta-actin-like protein (Q562R1). Deisotoped peptide ion m/z: 1066.55^{+1}.

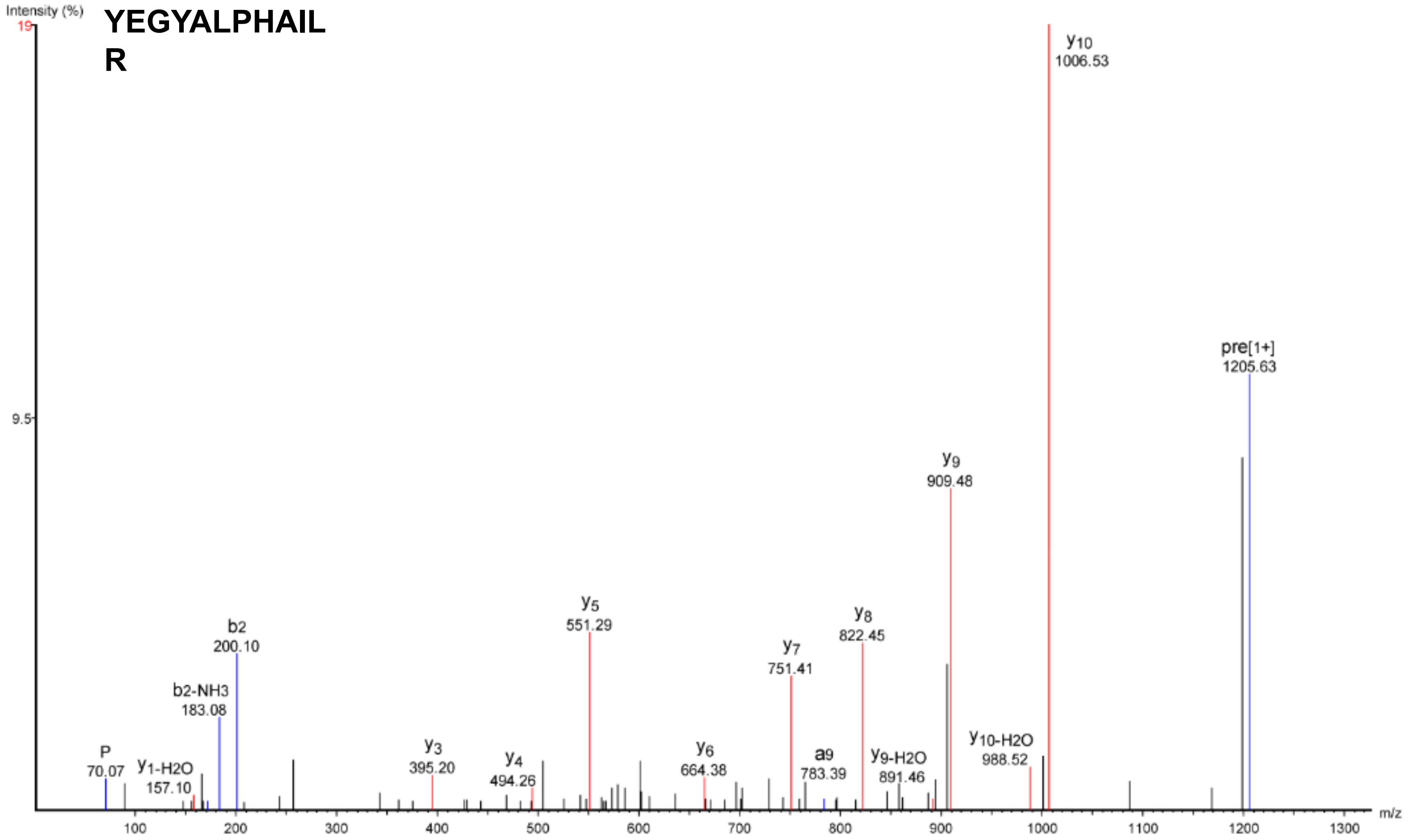

MS/MS spectrum of the peptide YEGYALPHAILR identified from beta-actin-like protein (Q562R1). Deisotoped peptide ion m/z: 1205.63^{+1}.

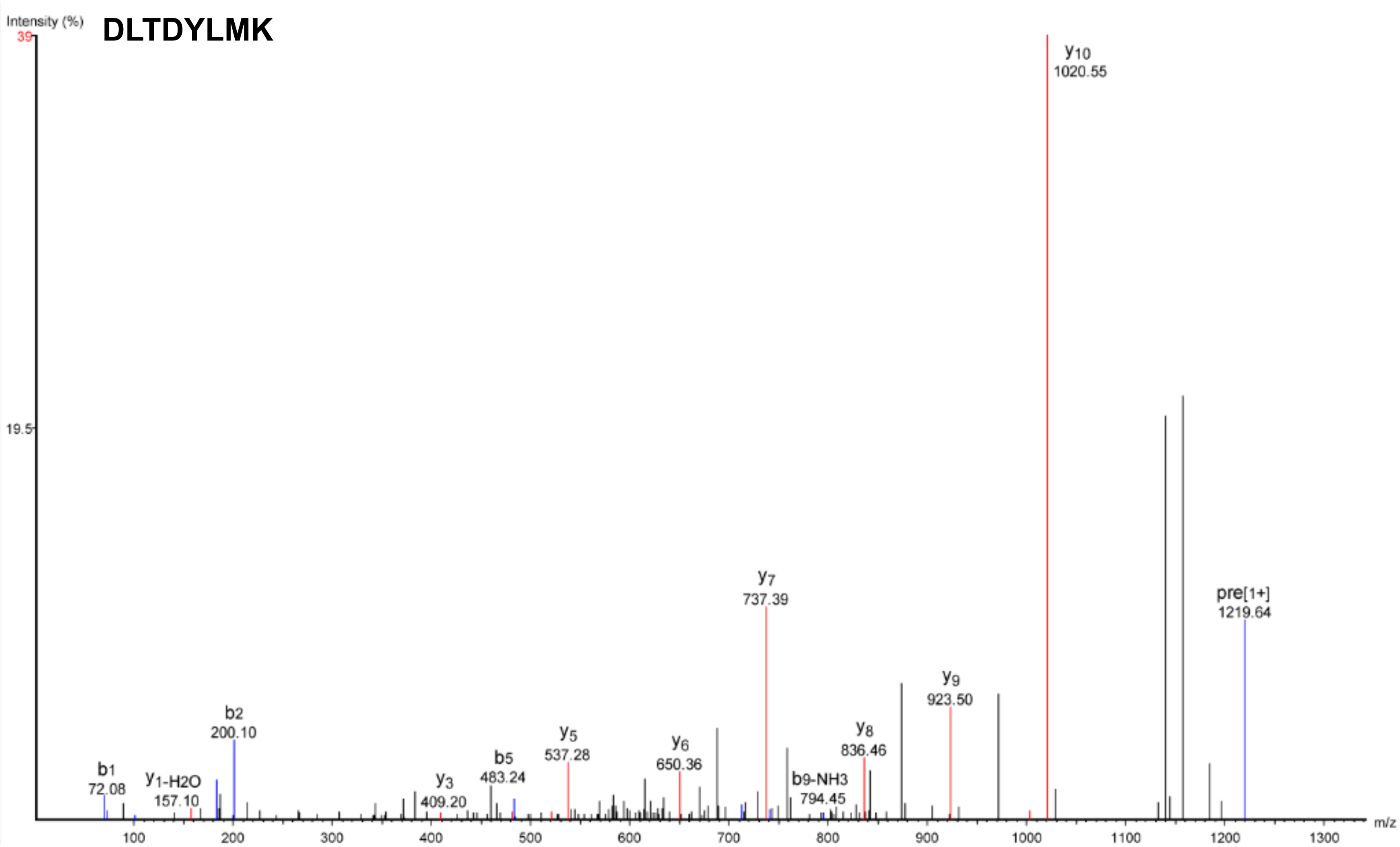

MS/MS spectrum of the peptide DLTDYLMK identified from beta-actin-like protein (Q562R1). Deisotoped peptide ion m/z: 1219.64^{+1}.

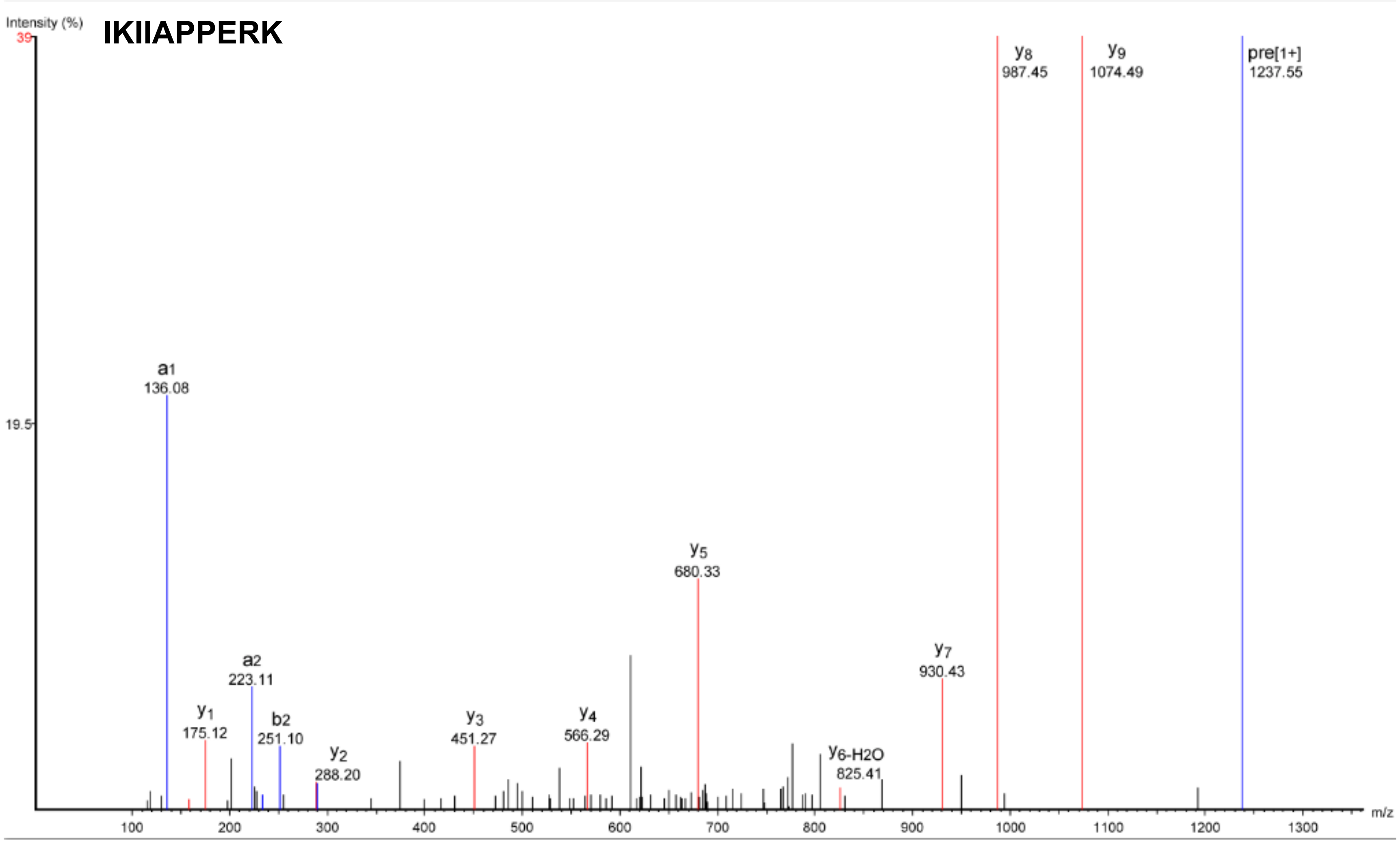

MS/MS spectrum of the peptide IKIIAPPERK identified from beta-actin-like protein (Q562R1). Deisotoped peptide ion m/z: 1237.55^{+1}.

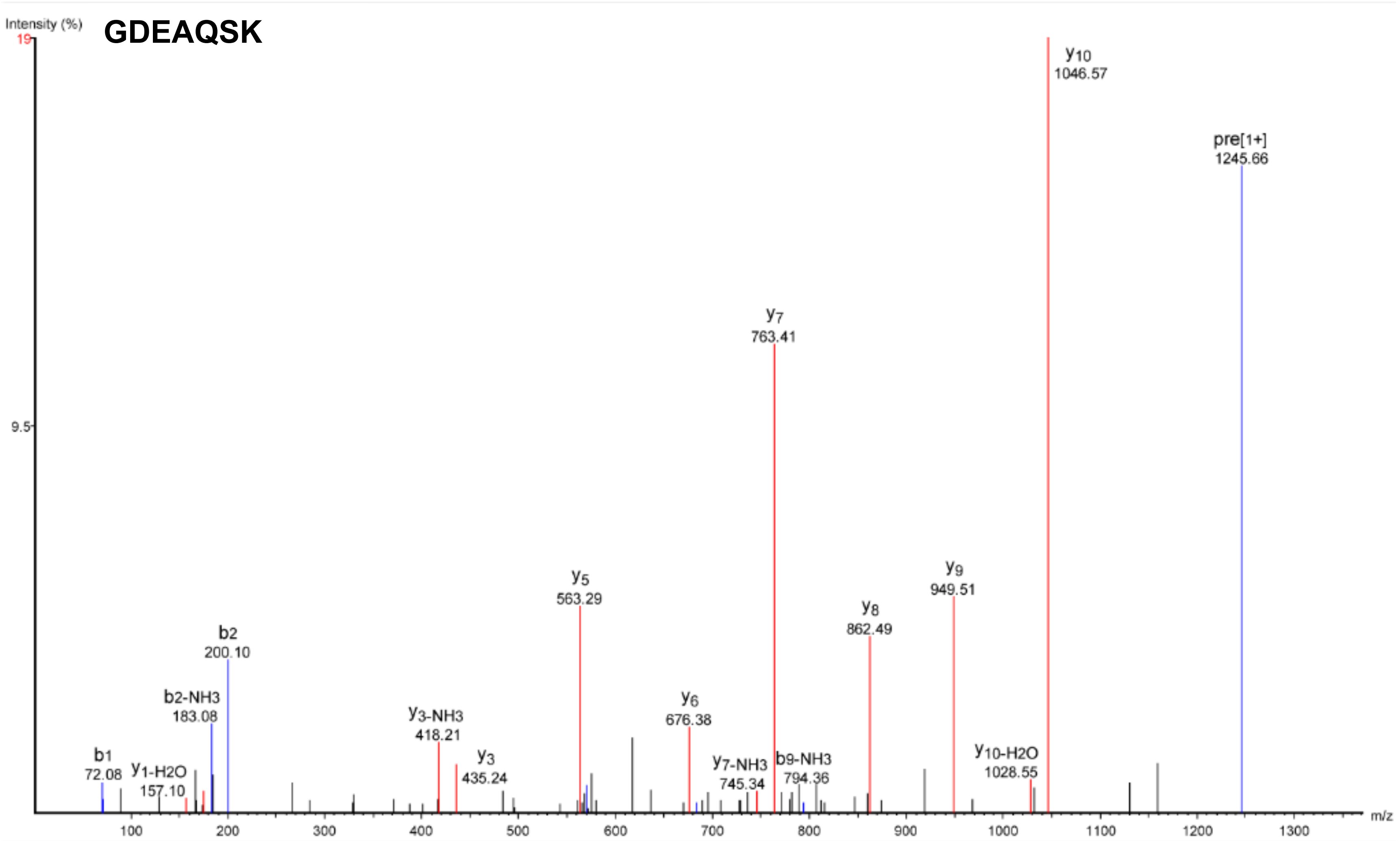

MS/MS spectrum of the peptide GDEAQSK identified from beta-actin-like protein (Q562R1). Deisotoped peptide ion m/z: 1245.66^{+1}.

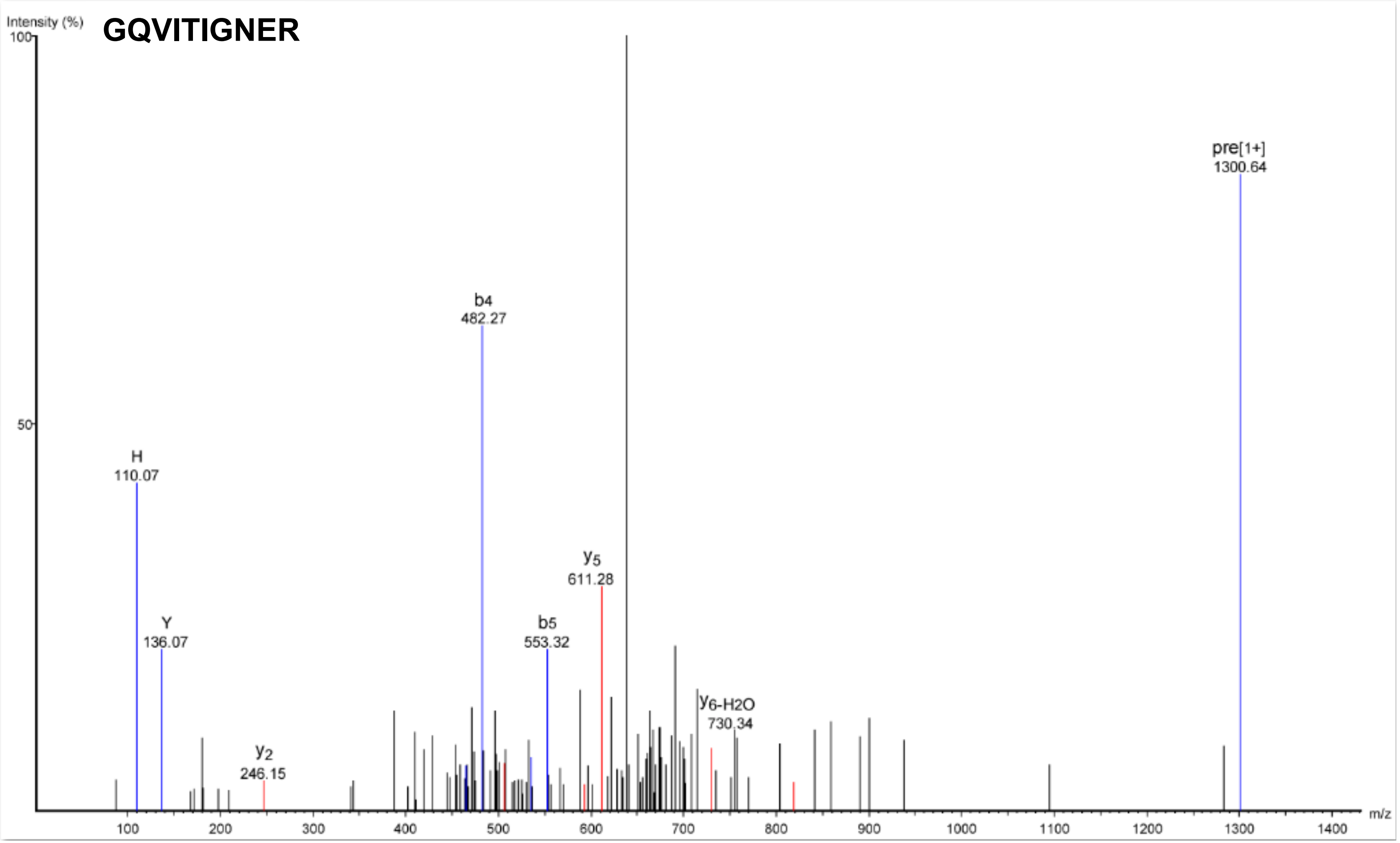

MS/MS spectrum of the peptide GQVITIGNER identified from beta-actin-like protein (Q562R1). Deisotoped peptide ion m/z: 1300.64^{+1}.

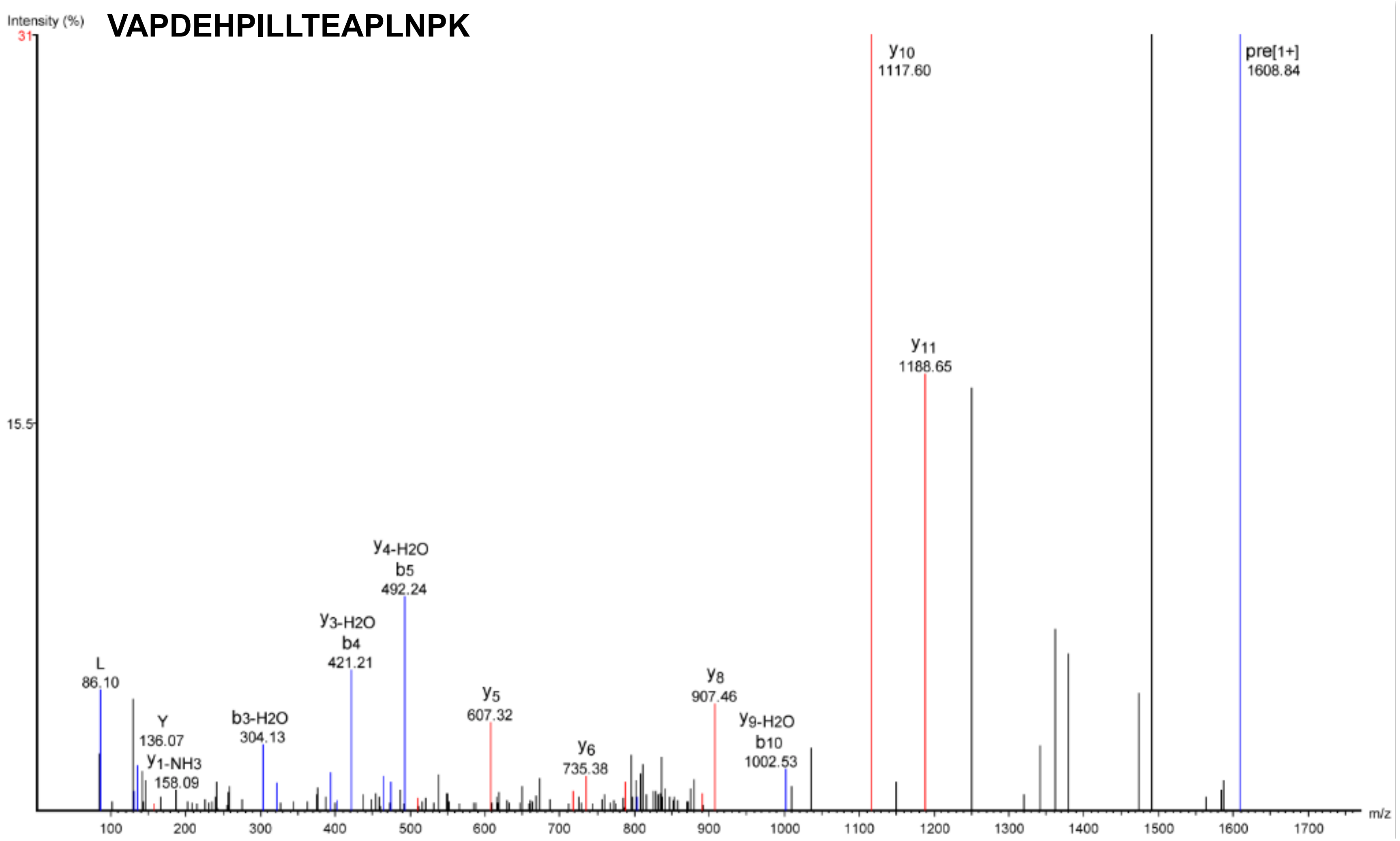

MS/MS spectrum of the peptide VAPDEHPILLTEAPLNPK identified from beta-actin-like protein (Q562R1). Deisotoped peptide ion m/z: 1608.84^{+1}.

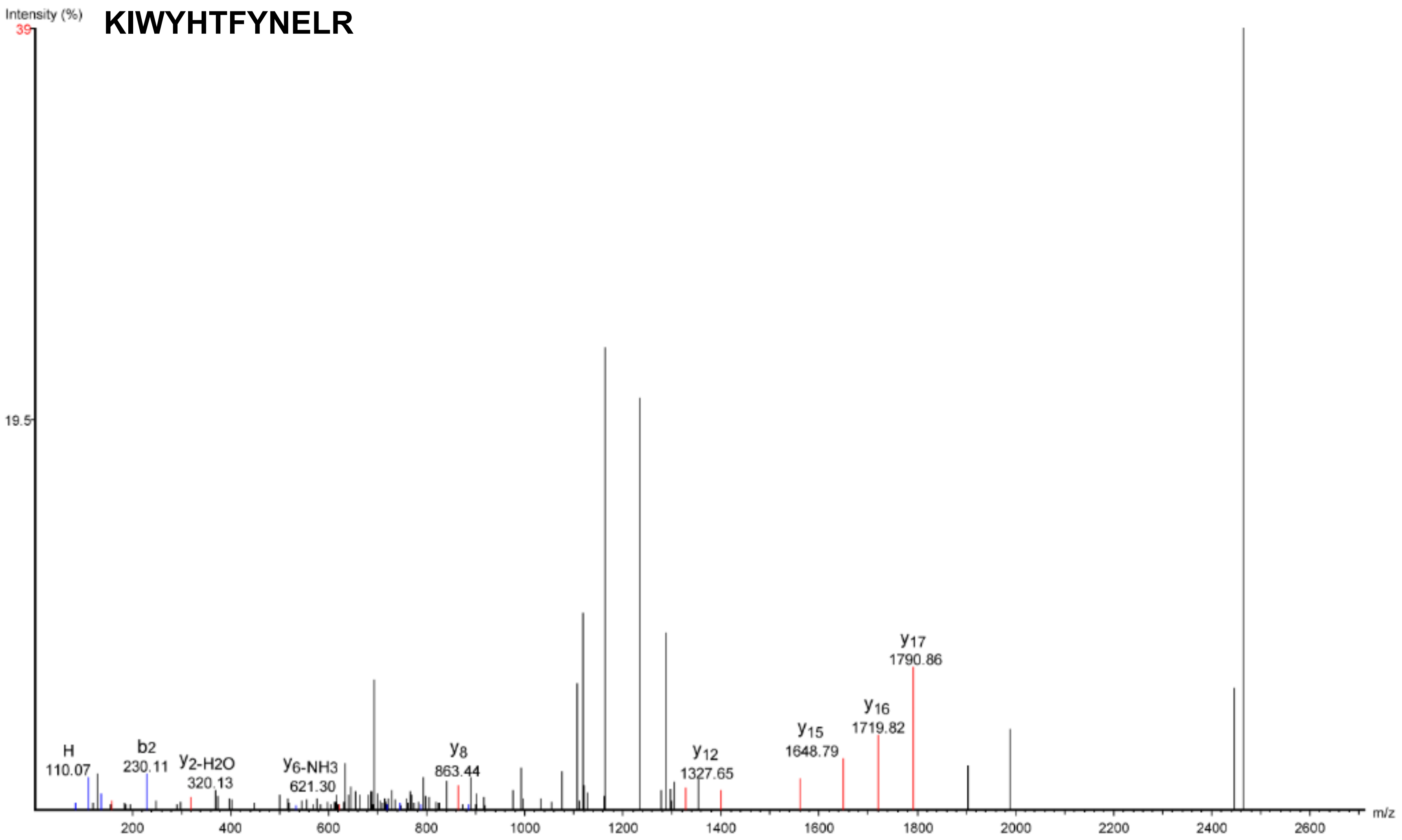

MS/MS spectrum of the peptide KIWYHTFYNELR identified from beta-actin-like protein (Q562R1). Deisotoped peptide ion m/z: 2465.18^{+1}.

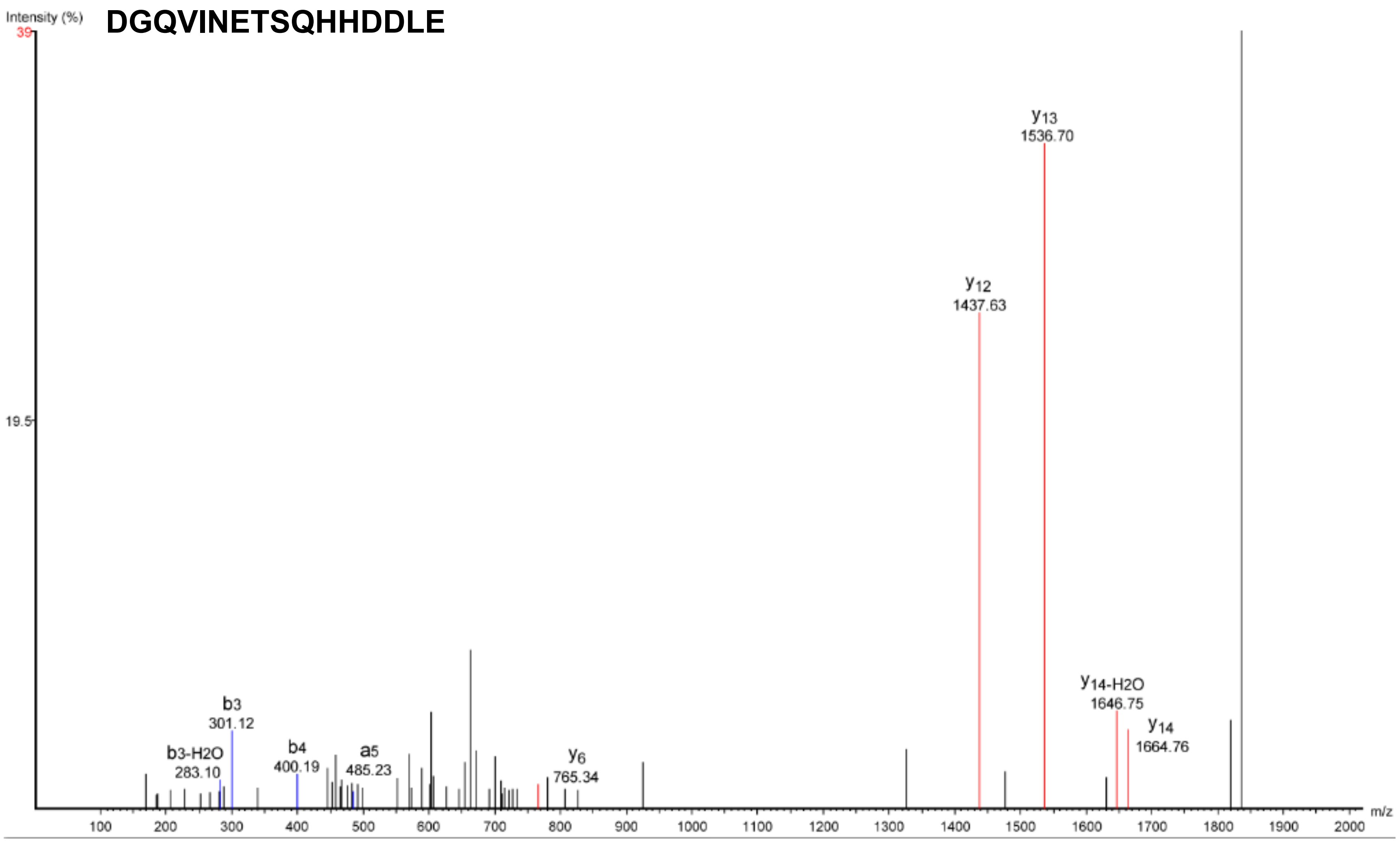

MS/MS spectrum of the peptide DGQVINETSQHHDDLE identified from human vimentin (P08670). Deisotoped peptide ion m/z: 1836.80^{+1}.

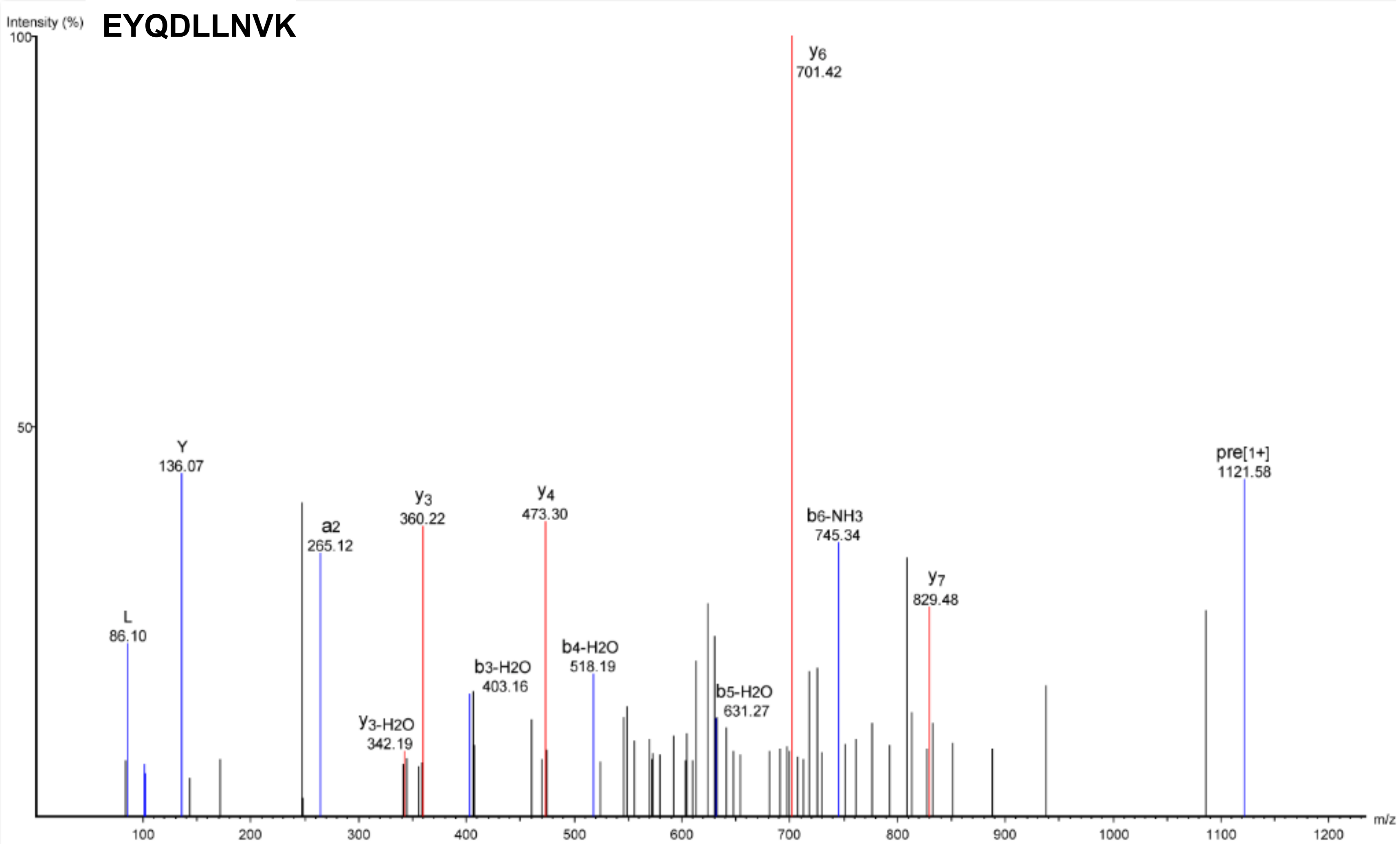

MS/MS spectrum of the peptide EYQDLLNVK identified from human vimentin (P08670). Deisotoped peptide ion m/z: 1121.58^{+1}.

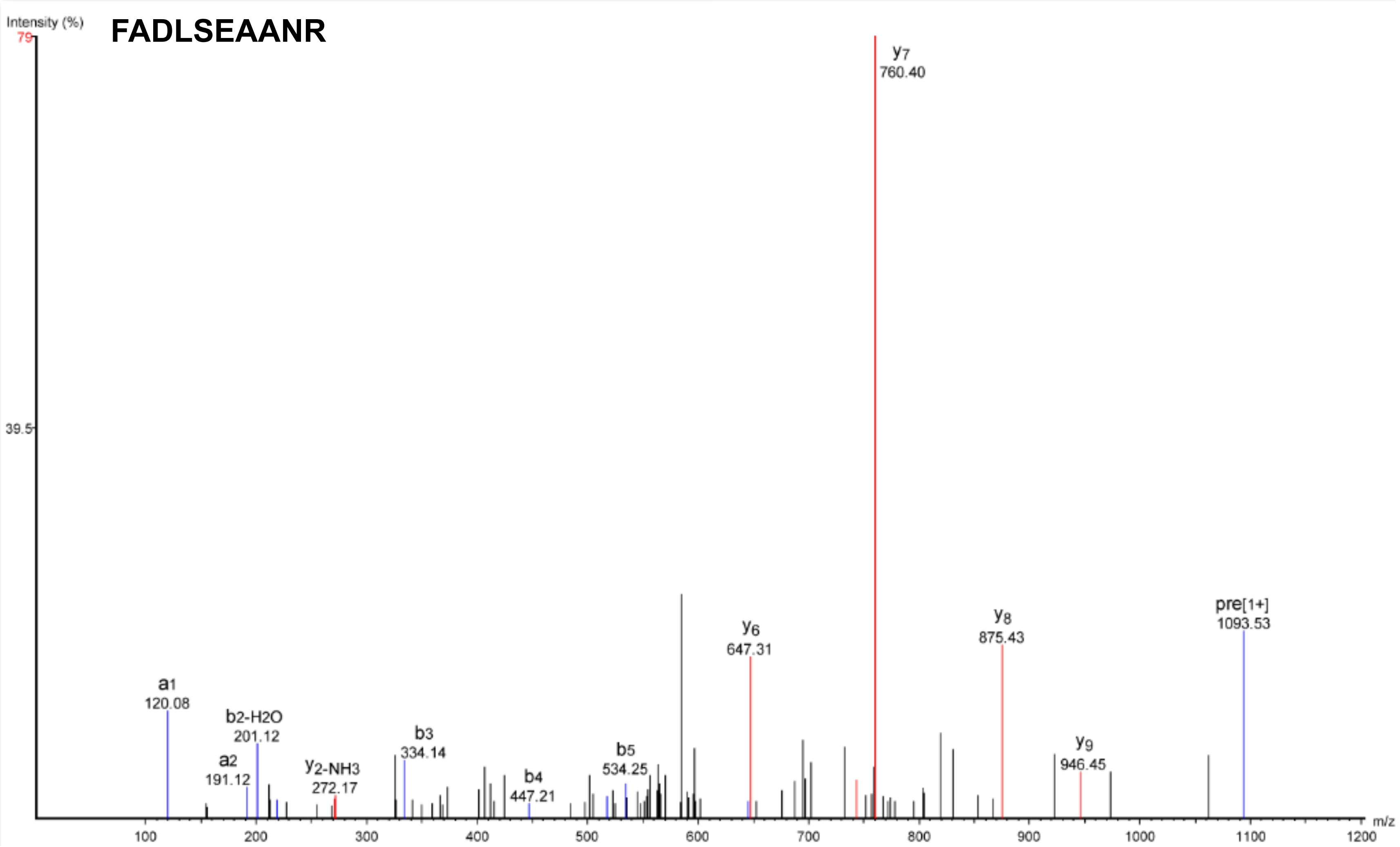

MS/MS spectrum of the peptide FADLSEAANR identified from human vimentin (P08670). Deisotoped peptide ion m/z: 1093.53^{+1}.

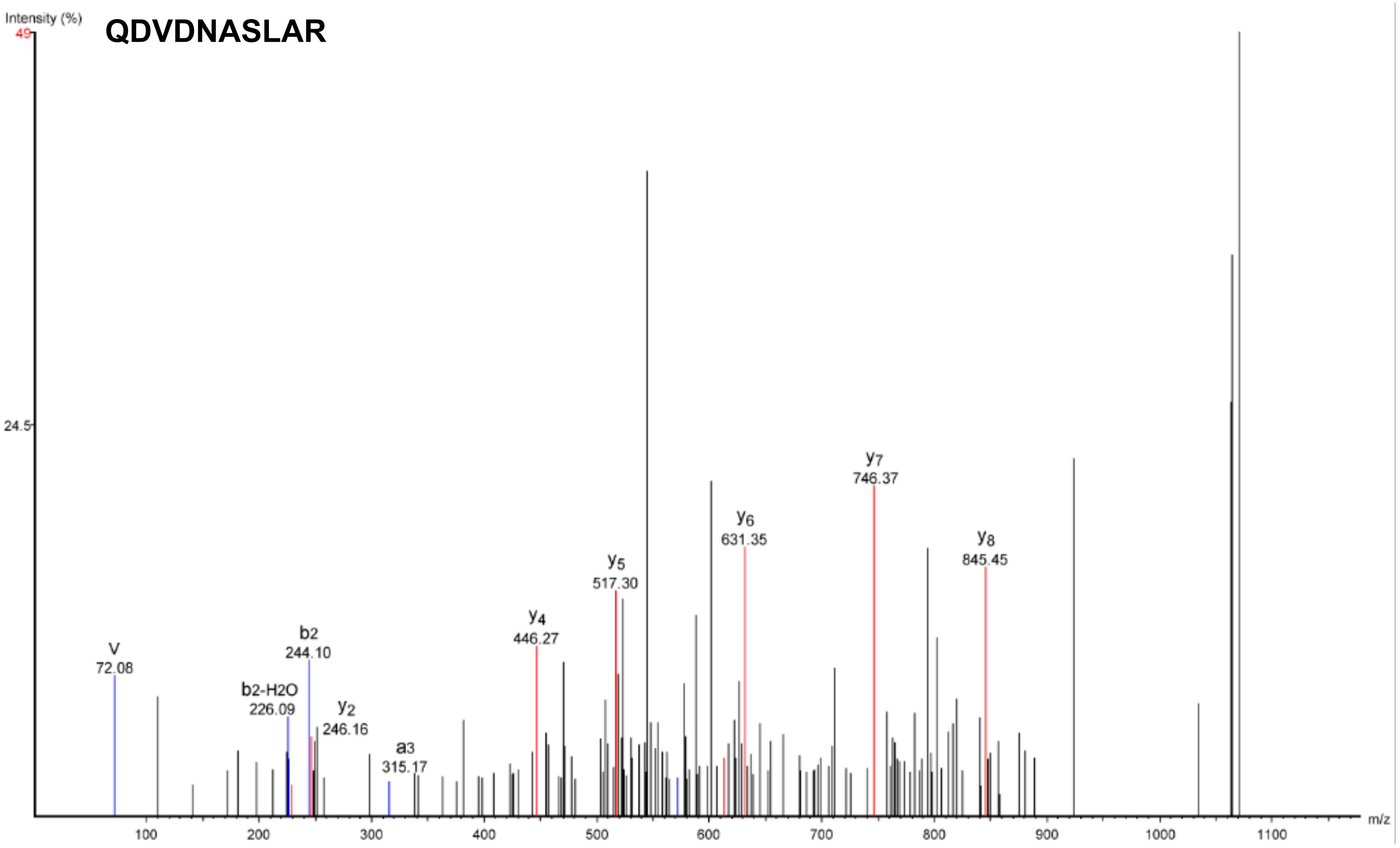

MS/MS spectrum of the peptide QDVDNASLAR identified from human vimentin (P08670). Deisotoped peptide ion m/z: 1088.53^{+1}.

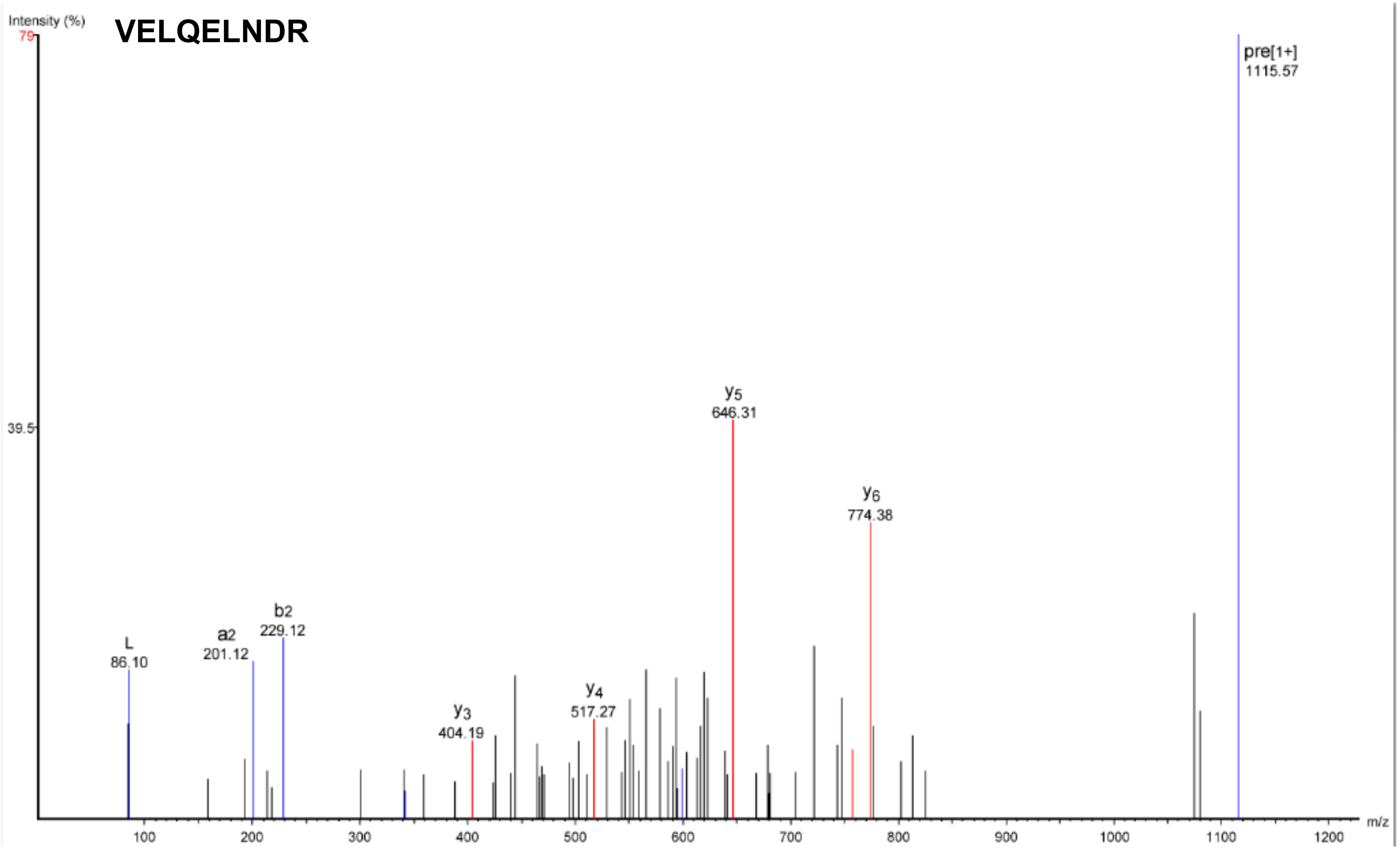

MS/MS spectrum of the peptide VELQELNDR identified from human vimentin (P08670). Deisotoped peptide ion m/z: 1115.57^{+1}.

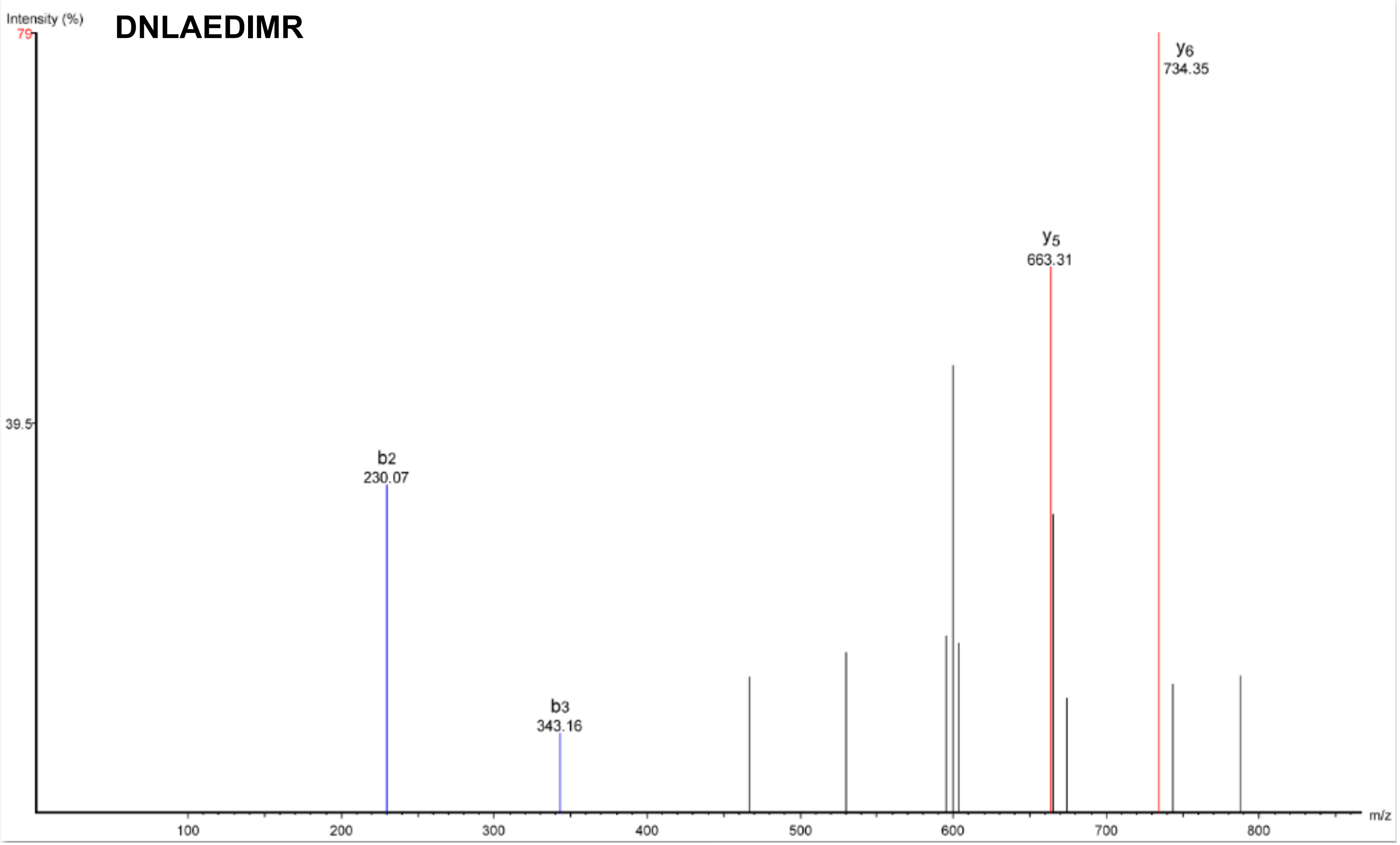

MS/MS spectrum of the peptide DNLAEDIMR identified from human vimentin (P08670). Deisotoped peptide ion m/z: 1076.50^{+1}.

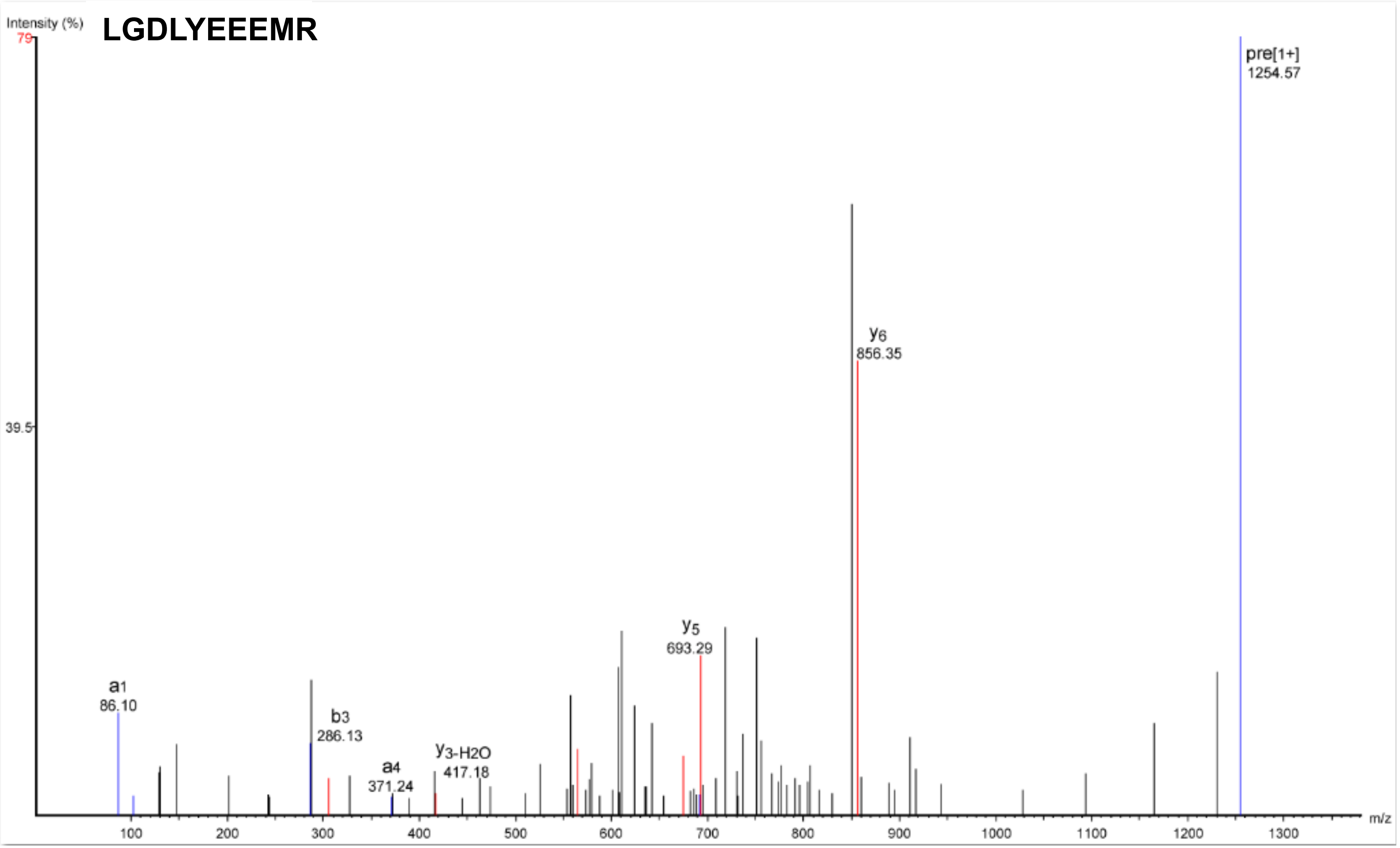

MS/MS spectrum of the peptide LGDLYEEEMR identified from human vimentin (P08670). Deisotoped peptide ion m/z: 1254.57^{+1}.

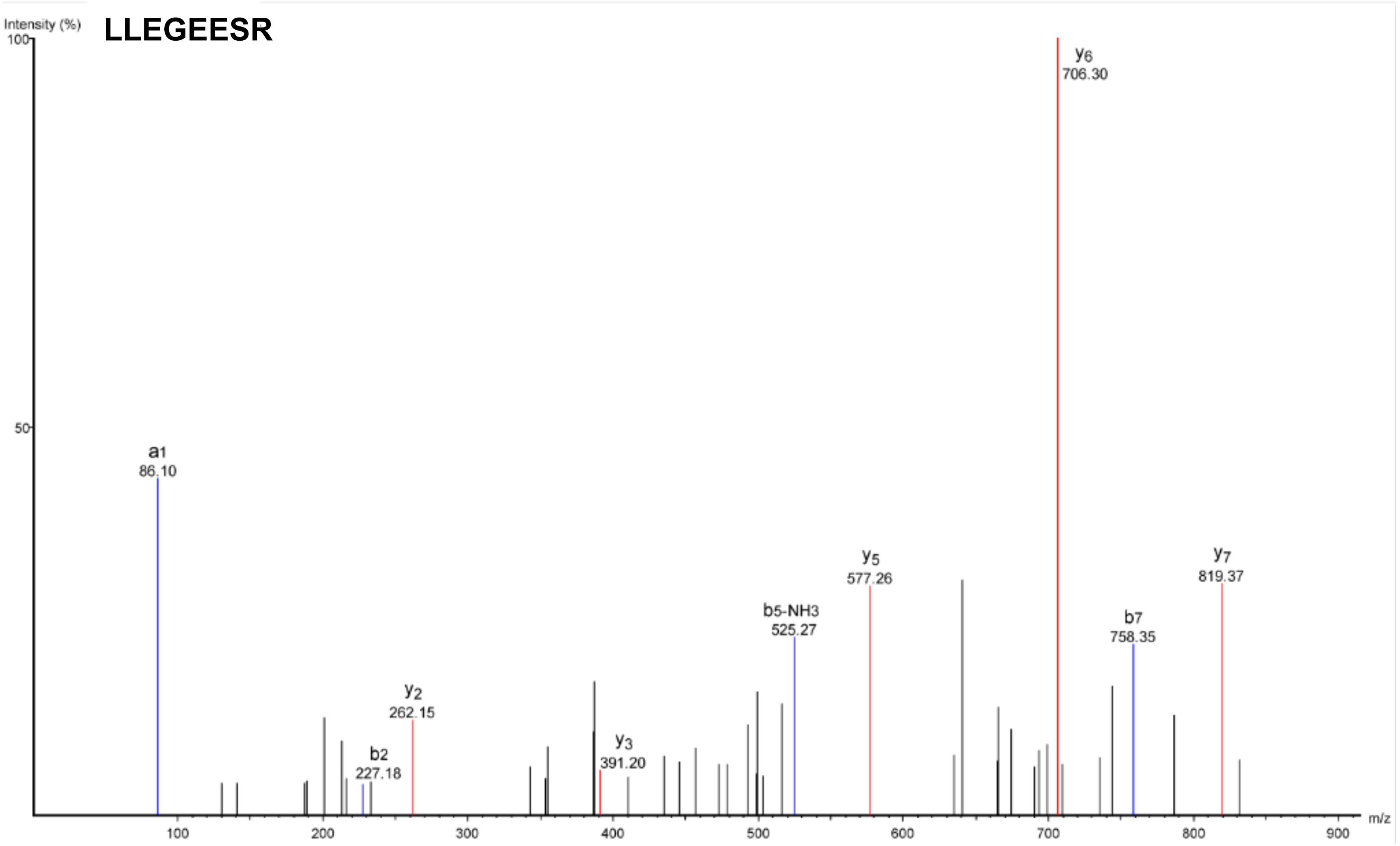

MS/MS spectrum of the peptide LLEGEESR identified from human vimentin (P08670). Deisotoped peptide ion m/z: 932.46^{+1}.
